# Predictive Model for Drug-Induced Liver Injury Using Deep Neural Networks Based on Substructure Space

**DOI:** 10.3390/molecules26247548

**Published:** 2021-12-13

**Authors:** Myung-Gyun Kang, Nam Sook Kang

**Affiliations:** 1 Department of Predictive Toxicology, Korea Institute of Toxicology, Daejeon 34114, Korea; myung-gyun.kang@kitox.re.kr; 2 Graduate School of New Drug Discovery and Development, Chungnam National University, Daejeon 34134, Korea

**Keywords:** drug-induced liver injury, DILI, deep neural network, DNN, ECFP4, in silico model, applicability domain, substructure space, endurance level, machine learning

## Abstract

Drug-induced liver injury (DILI) is a major concern for drug developers, regulators, and clinicians. However, there is no adequate model system to assess drug-associated DILI risk in humans. In the big data era, computational models are expected to play a revolutionary role in this field. This study aimed to develop a deep neural network (DNN)-based model using extended connectivity fingerprints of diameter 4 (ECFP4) to predict DILI risk. Each data set for the predictive model was retrieved and curated from DILIrank, LiverTox, and other literature. The best model was constructed through ten iterations of stratified 10-fold cross-validation, and the applicability domain was defined based on integer ECFP4 bits of the training set which represented substructures. For the robustness test, we employed the concept of the endurance level. The best model showed an accuracy of 0.731, a sensitivity of 0.714, and a specificity of 0.750 on the validation data set in the complete applicability domain. The model was further evaluated with four external data sets and attained an accuracy of 0.867 on 15 drugs with DILI cases reported since 2019. Overall, the results suggested that the ECFP4-based DNN model represents a new tool to identify DILI risk for the evaluation of drug safety.

## 1. Introduction

One of the major challenges for pharmaceutical industries and clinical researchers is to address safety concerns in humans [1]. According to recent studies, most drug failures happen due to the fact of safety issues, with approximately 25% of clinical failures in phase II and 14% in phase III, whereas 31% are associated with new drug or biological license application failures [2,3,4]. DILI is the main cause of acute liver failure (ALF) in the United States [5]. Drug-induced ALF accounts for approximately 20% of ALF in children and a higher percentage of ALF in adults [6]. Although the incidence rate of DILI is typically less than 1 in 100,000 to 1 in 10,000 patients, it is sometimes higher [6]. Importantly, DILI is one of the major causes of drug withdrawal from preclinical tests, clinical studies, and post-marketing stages [7,8]. DILI is usually characterized by the unexpected harmful effects that drugs exert on the liver, leading to damage of liver cells and other cells in the liver. DILI can be divided into two classes: intrinsic and idiosyncratic. Intrinsic DILI occurs in a dose-dependent manner, from a few hours to days after drug exposure. In contrast, idiosyncratic DILI is evoked by the combination of patient or environmental factors and drugs or drug metabolites [9], with a longer latency period from a few days to several months [10]. However, no specific in vivo, in vitro, or in silico model has been applied yet to predict the potential of new drug candidates to cause DILI in humans. Currently, the determination of the DILI risk of compounds, although quite challenging, is urgently required.

The FDA has published a reference drug list for DILI risk, called DILIrank, which is the largest annotated DILI data set [11]. It is composed of 1036 FDA-approved drugs that were defined from drug labels and an evaluation of causality evidence in the literature. This encouraged the development of computational models to predict DILI risk based on chemical structures. For DILI prediction, in silico approaches are attractive to researchers because they can have more benefits than in vitro or in vivo models in terms of time and expense. Therefore, many in silico models have been developed using their own characteristic descriptors and unique algorithms. Machine learning algorithms, such as Bayesian classification, support vector machine (SVM), and random forest (RF), have been used extensively for the development of predictive models of DILI risk. Liew et al. developed an ensemble model of 617 base classifiers to achieve an accuracy of 0.75, a sensitivity of 0.819, a specificity of 0.646, and an area under the receiver operating characteristic curve (AUC) of 0.595 for an external data set of 120 compounds [12]. Korsampasakou et al. produced a two-class prediction model for DILI based on an RF algorithm with 192 2D molecular descriptors [13]. Evaluation of the model showed an accuracy of 0.60, a sensitivity of 0.67, a specificity of 0.67, and an AUC of 0.64 for a merged test set of 966 compounds. Zhang et al. generated the best SVM-based model with an FP4 fingerprint, showing an accuracy of 0.75, a sensitivity of 0.932, a specificity of 0.379, and an AUC of 0.667 for an 88 external data set [14]. Ai et al. utilized SVM, RF, and extreme gradient boosting algorithms to create an ensemble model with 12 molecular fingerprints. The ensemble model achieved an accuracy of 0.843, a sensitivity of 0.869, a specificity of 0.754, and an AUC of 0.904 [15]. He et al. also built an ensemble model with eight algorithms for predicting the DILI risk of xenobiotics, and the model attained an accuracy of 0.773, a sensitivity of 0.658, a specificity of 0.730, and an AUC of 0.716 on an entire external data set that consisted of the three external data sets reported in Ai et al. [15], Zhang et al. [14], and Kortsampasakou et al. [13].

In this study, we obtained an approximately equal number of DILI-positive and DILI-negative drugs from the DILIrank and the LiverTox databases [16] to exclude the development of a biased model and prepared an extra data set for model validation. Although the data size was not sufficient for deep learning, we selected the deep neural network (DNN) algorithm for DILI classification, which was expected to contribute to a more accurate and reliable model, since new combinations of molecular features to discriminate DILI-positives and DILI-negatives would be created during model training processes owing to their ability to unearth complex features from high-dimensional data [17]. Moreover, to define the applicability domain of the developed model, we used integer ECFP4 fingerprint bits, each corresponding to a specific substructure. All integer bits extracted from the training data set were regarded as the applicability domain of the model, and the ratio of integer bits outside this domain was defined as the endurance level of each compound in the validation data set. By applying this concept to each data set, we could develop a more reliable model.

For model development with the training data set, stratified cross-validation [18,19] was performed to suppress overfitting, which is one of the principal issues in DNNs [20], and after ten iterations of cross-validation, the best model was chosen for testing loss. Additionally, to verify the usefulness of the model, four external data sets and 15 DILI case-reported drugs were retrieved and evaluated for DILI potential. The model developed in this study showed a fairly good performance, especially with respect to sensitivity, and its potential to identify compounds associated with a high DILI risk was proven.

## 2. Results

### 2.1. Preparation of Data Sets and Applicability Domain

For the development of the DILI-prediction model using a DNN algorithm, the retention of well-annotated DILI data sets is essential. Among the many DILI-related drug lists, the DILIrank data set, the LiverTox data set, the data set from Xu et al. (Xu data set) [21], and that from Greene et al. (Greene data set) [22] were chosen for this study. Following data preprocessing, such as the removal of mixtures, metal-containing drugs, and redundant compounds, the first two data sets were integrated into the training data set, and the remaining two data sets were used for validation (Table 1). In the DILIrank data set, drugs classified as vMost-DILI-Concern or vLess-DILI-Concern were considered DILI-positives and those classified as vNo-DILI-Concern were considered DILI-negatives [23]. In the LiverTox data set, drugs with a likelihood score of “A” or “B” were grouped as DILI-positives, while those with a score of “E” or “E*” were grouped as DILI-negatives. All compounds in the Xu data set were already classified as DILI-positives or DILI-negatives. The Greene data set categorized the compounds into four classes; only drugs in the classes of “no evidence (NE)” and “human hepatotoxicity (HH)” were chosen and classified as DILI-negatives and DILI-positives, respectively. Overall, the training data set consisted of 479 DILI-negatives and 461 DILI-positives, and the validation data set included 74 DILI-negatives and 105 DILI-positives.

**Figure 1:**
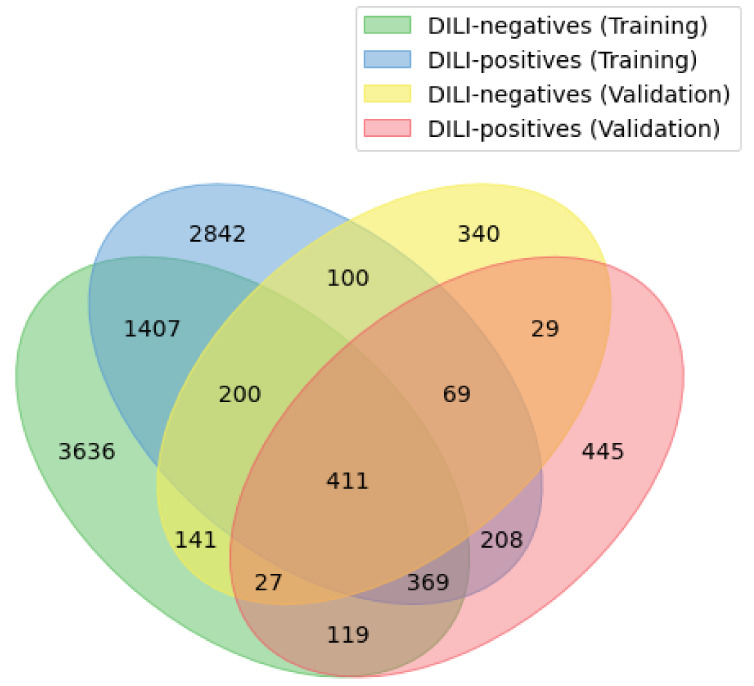
Venn diagram of unique integer ECFP4 fingerprint bits in the training data set and validation data set.

To define the applicability domain of the model (see Section 4), integer ECFP4 bits were used. In the training data set, 6310 unique bits were found in DILI-negatives and 5606 were found in DILI-positives, and the number of unique substructures shared between the two classes was 2387. However, in the validation data set, DILI-negatives had 1317 unique substructures, DILI-positives had 1677 unique substructures, and the two classes were found to share 536 unique substructures. The number of substructures that occurred in the validation data set but not in the training data set was 814 (Figure 1). The most prevalent substructures in the validation data set were “CC(N)C(=O)[O–]” and “CN(C)C(C(=O)[O–])C(C)(C)S”, both of which were found in six compounds, namely, azlocillin sodium, cloxacillin sodium, mezlocillin sodium, methicillin sodium, and dicloxacillin sodium, which are DILI-positive, and penicillin G sodium, which is DILI-negative. The second most-frequent substructures were six in number and identified in four drugs for each substructure. Specifically, “ccn(cn)CC” was detected in sulconazole nitrate, miconazole, butoconazole nitrate, and econazole nitrate, whereas “CNCCN”, “CCNC(C)C”, “CC(N)CN(C)c”, “CC(N)CN(c)C”, and “CNC(C)CN” were found in lomefloxacin HCl, gatifloxacin, temafloxacin, and grepafloxacin. In addition, 15 substructures were identified in three compounds for each, and 53 substructures were discovered in two compounds per substructure. The full list of substructures is shown in Table S1.

### 2.2. Development and Validation of DNN-Based DILI-Prediction Model

We trained the DNN model for DILI prediction with stratified 10-fold cross-validation, which is commonly used to avoid the overfitting problem for a small data set by partitioning data to reduce variability [24]. To create the best DNN model, four fingerprints, namely, the extended connectivity fingerprint 4 (ECFP4), ECFP6, functional class fingerprint 4 (FCFP4), and FCFP6 were tested and compared. Each descriptor was separately applied, and each model was generated through ten iterations of cross-validation. The best model was selected based on the loss values obtained by testing the holdout test set. The best model for ECFP4 was generated at iteration 6of the cross-validation, with a loss value of 0.0837 and a mean accuracy of 0.940 ± 0.0859 (Table 2 and Table S2). ECFP6 developed the best model with a loss value of 0.0768 and a mean accuracy of 0.958 ± 0.0768 at iteration 3. The loss value and mean accuracy for the best model created with the two FCFP fingerprints were 0.0566 and 0.952 ± 0.0837, respectively, at iteration 6 for FCFP, and 0.0516 and 0.948 ± 0.0748 at iteration 4 for FCFP6 (Table S2).

**Figure 2:**
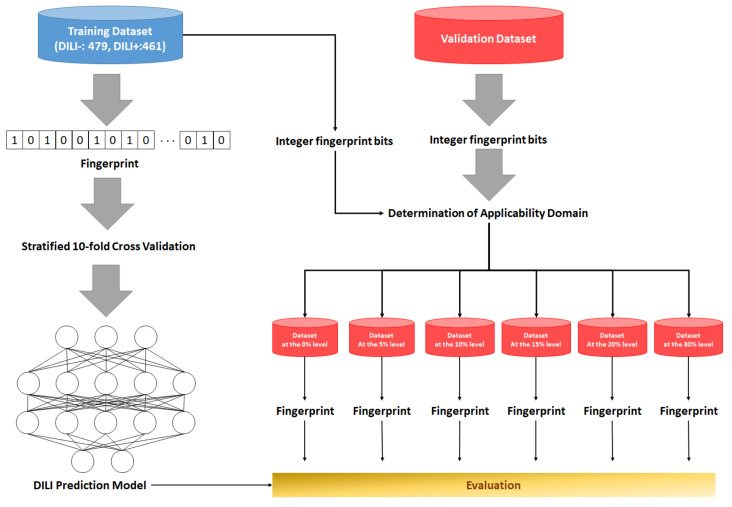
Overall workflow for model creation and evaluation. Models were created based on chemical structure-based fingerprints using various algorithms and subsets from validation data sets selected by the applicability domain, which was defined by the endurance level.

The performance of the models was assessed based on the metrics of accuracy, sensitivity, specificity, and F1 score with subsets of the validation data set that were selected at a variety of endurance levels, which were defined by integer bits of each fingerprint (Table 3). The ECFP-based models showed a better predictive performance than FCFP-based models in most of the measured metrics. The ECFP6-based model had slightly higher specificity values than the ECFP4-based model. However, the ECFP4-based model showed a better performance in all metrics except for specificity, although it attained slightly lower accuracies at the 0% and 5% levels. The ECFP4-based model achieved the best sensitivity of 0.778 at the 5% level, and the sensitivities were sustained above 0.71 across all endurance levels. Overall, the accuracies of the model varied from 0.607 (30%) to 0.731 (0%), the sensitivities varied from 0.714 (0%) to 0.778 (5%), the specificities varied from 0.397 (30%) to 0.750 (0%), and the F1 score varied from 0.686 (30%) to 0.741 (0%). The most outstanding performance of the ECFP4-based model was achieved in the absolute applicability domain (at an endurance level of 0%), based on which we determined the ECFP4 model to be the best DNN model and used it for further evaluation. As the endurance level increased, the metrics tended to decrease, thereby supporting the fact that the applicability domain was well established and could not contribute to the assessment of the model. The overall process of generating the best DNN model is illustrated in Figure 2.

**Figure 3:**
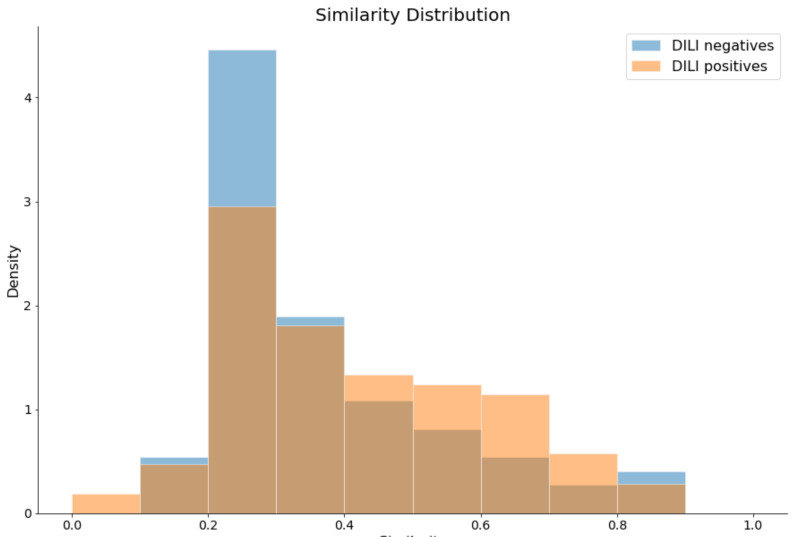
Histogram of maximum similarities for each DILI class. The graph indicates that the DILI-negatives in the validation data set had a more biased distribution of the maximum similarity, ranging from 0.2 to 0.3, than DILI-positives in the same data set. This indicates that the DILI-negatives structurally varied more than the DILI-positives.

When the models were tested with the validation data set, they showed high sensitivities but moderate accuracies. This was attributable to relatively low specificities; we hypothesized that these patterns could arise from the greater structural diversity of DILI-negatives than of DILI-positives. To prove this, we calculated the Tanimoto distance of the ECFP4 fingerprint between each of the same classes in the training data set and validation data set (Figure 3 and Table S3). For each compound in the validation data set, the maximum similarity with compounds in the same class of the training data set was calculated. The average similarity between DILI-negatives was 0.363 ± 0.1712, and that between DILI-positives was 0.414 ± 0.1866. The statistical difference between the two similarities was proven to be significant, with a *p*-value of 0.0319 in a one-tailed *t*-test. Therefore, these results demonstrated that the DILI-negatives were more diverse than the DILI-positives in terms of chemical structures.

### 2.3. Performance Comparison of the Best DNN Model with Machine Learning Models

We then compared the performance of the ECFP4-based DNN model with that of representative machine learning (ML) models such as the Bernoulli naive Bayesian (NB), SVM, and RF models (Table 4). Model performance was evaluated based on accuracy, sensitivity, specificity, and the F1 score. Except for the fact that the NB model achieved the best specificities (0.767–0.810) at endurance levels ranging from 5% to 30%, the DNN model showed better predictive results than the three tested ML models in all metrics at all endurance levels. It is especially notable that no ML model showed a better performance than the DNN model in the complete applicability domain. In contrast, the NB model had the lowest specificity, and the SVM and RF models showed a moderate performance. These results proved that the DNN model can be superior to ML models at the single model level.

### 2.4. Performance Test of the DNN Model on the External Data Sets from Prior Studies

Next, we tested the performance of the DNN model on four external data sets that were tested in previous studies [12,13,14,15]. The compounds to be tested in each external data set were newly chosen by endurance levels. Table 5 shows the test results for compounds within the 0% and 100% levels; the whole test results are shown in Table S4.

The validation data set from Liew et al. [12] (Liew data set) was composed of three kinds of data sets: valBLACK, valPAIR, and valRANDOM. The valBLACK contains 23 drugs withdrawn from the market or with a black box warning for hepatotoxicity and 24 nontoxic compounds. The valPAIR consists of ten pairs of structurally similar compounds which, however, exhibit different toxicities. valRANDOM, which consists of 48 negative and 72 positive compounds, was generated through stratified sampling of the pre-trained data. By applying the endurance level of 0%, the valBLACK data set decreased to 38, the valPAIR data set to 14, and the valRANDOM data set to 62 compounds. For performance comparison, validation results generated by the five-fold cross-validated kNN (k = 9) model were chosen, because the model was referred to as the best-performing model in Liew et al. [12]. As compared to the model of Liew et al. [12], our model accomplished better predictive results with respect to all metrics, except for the specificity for valPAIR and valRANDOM at an endurance level of 0%. However, the metrics measured at an endurance level of 100% were similar to or lower than those reported in Liew et al. [12]. In addition, the model performance on the entire Liew data set also showed better results at the 0% than at the 100% level.

The validation data set from Zhang et al. [14] (Zhang data set) was collected from the liver toxicity benchmark database of the FDA named the Liver Toxicity Knowledge Base (LTKB) [8]. Zhang’s data set consisted of 28 DILI-negatives and 57 DILI-positives. Among them, 80 compounds (53 DILI+ and 27 DILI−) were identified within an endurance level of 0%, and the test results showed an accuracy of 0.950, a sensitivity of 1.000, a specificity of 0.926, and an AUC of 0.957. At the 100% level, our model achieved similarly remarkable predictive results compared to Zhang’s model based on the SVM method, while our model had a better predictive ability, especially regarding specificity and AUC.

The external validation data set of Ai et al. [15] (Ai data set) was also prepared from LTKB and contained 221 DILI-positives and 65 DILI-negatives. After deletion of drugs that appeared in the training data set, a total of 121 compounds remained, and 84 compounds were collected at the 0% endurance level. The test results on these data sets also proved that the predictive performance of our model was slightly better than that of the ensemble model from Ai et al. [15]. Specifically, the increased specificities of 0.810 (at the 0% level) and 0.852 (at the 100% level) compared to 0.754 in the Ai data set were thought to contribute to better accuracies (0.881 at the 0% level and 0.893 at the 100% level compared to 0.843).

The external validation data set of Kotsampasakou et el. [13] (Kotsampasakou data set) that was collected from three data sources (Liew et al. [12], Chen et al. [11], and Mulliner et al. [25]) had 541 DILI-positives and 455 DILI-negatives. After removal of drugs duplicated with the training data set, 973 compounds (524 positives and 449 negatives) were retained, and after applying the endurance level of 0%, 151 compounds (84 DILI-positives and 67 DILI-negatives) were selected. As a test result, our model achieved a better performance with respect to accuracy, specificity, and AUC but a lower sensitivity than the Ai model at an endurance level of 0%. However, our model showed a slightly lower performance at an endurance level of 100%.

These comparisons demonstrated that the DNN-based model had a better performance than the referred to ML-based models. Moreover, the applicability domain defined by the endurance level of integer ECFP4 bits contributed to significantly enhance model performance.

### 2.5. DILI Prediction on Drugs That Have Case Reports on Liver Injury

Next, we evaluated the DNN model using drugs that were not included in the data set during data set preparation but had case reports related to DILI since 2019 when the DILI rank data set was released. Although it is risky to classify drugs as DILI-positives based on case reports only, they were nevertheless regarded as DILI-positives in this study. In total, 15 drugs were collected by a literature survey and tested for DILI risk using the pre-trained DNN model. The prediction results for the drugs are listed in Table 6. All drugs were present within an endurance level of 10%. We classified drugs as DILI-positive if the prediction probability was over 0.5; otherwise, drugs were classified as DILI-negative. Overall, all drugs except two, namely, ulipristal acetate and nilotinib, were classified as DILI-positive by the model; thus, test accuracy (sensitivity) for the external data set was calculated to be 0.867, and 60% (nine drugs) of the whole number of drugs showed a higher prediction probability than 0.9. These results implied that our model had a good predictive performance to accurately identify DILI drugs.

## 3. Discussion

The first prerequisite for a deep learning model is a sufficient number of data sets with a wide variety of properties to make the model work accurately [41]. For this reason, we retrieved the drug lists from two rich data sources that contained >1000 drugs, namely, the DILI rank data set [11] that uses FDA drug label information and the LiverTox data set [16] that is based on clinical evidence, and assigned them to the training data set. Two other data sources that have frequently been referenced in the literature [23], namely, the Xu [21] and Greene data sets [22], were utilized for the validation data set. Deep learning-based models are subjected to the generation of complicated feature extraction through intensive yet complex encoding layers and can produce a skewed model due to the unbalanced training data [42,43,44,45]. To overcome this, we aimed to prepare the same number of compounds for each DILI class and acquired a training data set that consisted of 479 DILI-positives and 461 DILI-negatives.

For a more reliable prediction, we newly defined the applicability domain of our models using integer ECFP4 bits. The domain was determined as the entire pool of substructures in the training data set. The endurance level for a drug indicates the ratio of substructures not defined in the domain. This concept was applied to all validation data sets to retrieve subsets acceptable to each endurance level, and we demonstrated the usefulness of this concept by showing that the best performance of our model was achieved at an endurance level of 0% for our validation data sets, and the Liew, Zhang, and Kotsampasakou data sets (Table 5). This was also true for other fingerprint-based models (Table 3). This new method to define an applicability domain is expected to improve model performance.

Overall, our model tended to perform better with respect to sensitivity than specificity, especially for our validation data set. One of the reasons for this might be that DILI-negatives have a more diverse chemical structure than DILI-positives. We proved this through the comparison of the maximum similarity between the two classes using a one-tailed *t*-test with the Tanimoto distance of ECFP4 fingerprints. The results showed that DILI-negatives were less similar than DILI-positives. This indicates that our model lacked coverage for DILI-negatives, and newer and more diverse DILI-negatives need to be added to the training data set to strengthen our model.

To further confirm the performance of the DNN model, we created a new external data set composed of 15 DILI drugs with case reports, based on a literature search covering the most recent three years (Table 6); the drugs were flucloxacillin (brand name: Floxapen (Actavis UK, UK), antibiotics), apixaban (brand name: Eliquis (Bristol-Myers Squibb, New York, NY, USA), anticoagulant), aliskiren (brand name: Tekturna (Novartis, Switzerland) and Rasilez (Novartis, Switzerland), anti-hypertension drug), olmesartan (brand name: Benicar (Daiichi Sankyo, Tokyo, Japan), anti-hypertension drug), levothyroxine (brand name: Levothroid (Lloyds Pharmaceutical, UK), synthetic thyroxine), phenprobamate (brand name: Carisoprodol (Mylan Specialty LP, Morgantown, WV, USA), skeletal muscle relaxant), pirfenidone (brand name: Esbriet (Genentech, South San Francisco, CA, USA), immunosuppressants), escitalopram (brand name: Lexapro (Allergan, Dublin, Ireland) and Cipralex (Sandoz, Switzerland), antidepressant drug), ligandrol (not approved), rilpivirine (brand name: Edurant (Janssen, Belgium) and Rekambys (Janssen, Belgium), HIV drug), zanubrutinib (brand name: Brukinsa (BeiGene, Cambridge, MA, USA), antineoplastic agents), fasiglifam (antidiabetic drug), mesterolone (brand name: Proviron, an androgen and anabolic steroid), ulipristal acetate (brand name: Ella (HRA Pharma, France), a medication used for emergency contraception), and nilotinib (brand name: Tasigna (Novartis, Switzerland), a medication for chronic myelogenous leukemia). A few case reports could not verify the drugs as DILI-positive; nevertheless, they were considered to have DILI potential and, hence, all drugs were treated as DILI-positives. These drugs showed limited diversity within the 10% endurance level, and a maximum level of 7.6% was observed with nilotinib. Although they had various indications, the DILI test results demonstrated that, except for ulipristal acetate and ligandrol, all drugs were predicted correctly as DILI-positives with a mean accuracy of 0.867. In particular, three of the 15 drugs, namely, fasiglifam, ulipristal acetate [46], and flucloxacillin [47], showed liabilities for idiosyncratic DILI. Fasiglifam was suggested to be idiosyncratic due to the delayed increase in alanine aminotransferase in some patients [48]. For ulipristal acetate, it was due to the structural features shared with telapristone acetate [49] and onapristone [50], which had been demonstrated to be idiosyncratic. Flucloxacillin HLA-B*57:01 is indicated as a clinical risk factor for idiosyncratic DILI [47]. However, the DNN model identified only fasiglifam and flucloxacillin as DILI-positives; the DILI properties of the two drugs might arise from their structures, although there has been no supportive report in that regard. Considering that the DNN model was built with the structural features of drugs, the results simply implied that hidden structural factors might cause idiosyncratic DILI.

The DNN model developed in this study exhibited a fairly high performance, especially for compounds that share structural properties with those of the training data set. For some drugs for which DILI cases have been reported recently, the model demonstrated an outstanding prediction capability. Therefore, the DNN model could potentially be used as a screening tool to identify and eliminate compounds that have DILI-causing potential in early stages of drug development and to promote drug discovery in the pharmaceutical industry ensuring human safety.

## 4. Materials and Methods

### 4.1. Data Set Preparation and Curation

The compounds with DILI annotation for humans were collected from four public sources: the DILIrank [11], LiverTox [16], Xu [21], and Greene data sets [22]. The first two were integrated into the training data set, and the other two were used as validation data sets. All individual data sets were combined into a single database. The CID of all drugs was updated by matching compound names from PubChem [51] using the Python package PubChemPy (https://pubchempy.readthedocs.io/en/latest/index.html, version 1.0.4). When multiple CIDs were assigned to one compound, the first one was selected. When the class labels were conflicting, the DILIrank label or LiverTox label was assigned. After retrieving the canonical SMILES for each drug based on their CIDs, all mixtures, compounds containing metals, and compounds without SMILES structures were eliminated. If any, ions and salts were removed from the compound structures. Duplicate compounds were removed from only the LiverTox, Xu, or Greene data sets. After preprocessing, a training data set consisting of 479 DILI-negative and 461 DILI-positive drugs was created, and the external data set had 74 DILI-negatives and 105 DILI-positives. Additionally, an external test data set was prepared from a literature search; it was composed of 12 drugs with at least one clinical case report of DILI since 2019. All were regarded as DILI-positive (Table 1). The four external validation data sets were prepared through removal of compounds that have the same SMILES structures or the same PubChem ID with the training data set. The PubChem ID for the validation data set were retrieved from PubChem [51] with their SMILES using PubChemPy.

### 4.2. Model Development and Architecture of the DNN Model

An ECFP4 of 1024 bits was used as the molecular descriptor for all drugs in the data sets. Fingerprints were obtained from the “GetMorganFingerprintAsBitVect” function with a radius parameter of 2 in the RDKit library (https://www.rdkit.org, version 2021.03.4) of Python (version 3.9.5). To prevent the weights of zero bits from not being updated during the model training, all 0 bits were changed to −1 bit. Each DILI label of the drugs was converted to a one-hot encoded vector of size 2, where the first bit was for DILI-negatives and the second bit was for DILI-positives.

**Figure 4:**
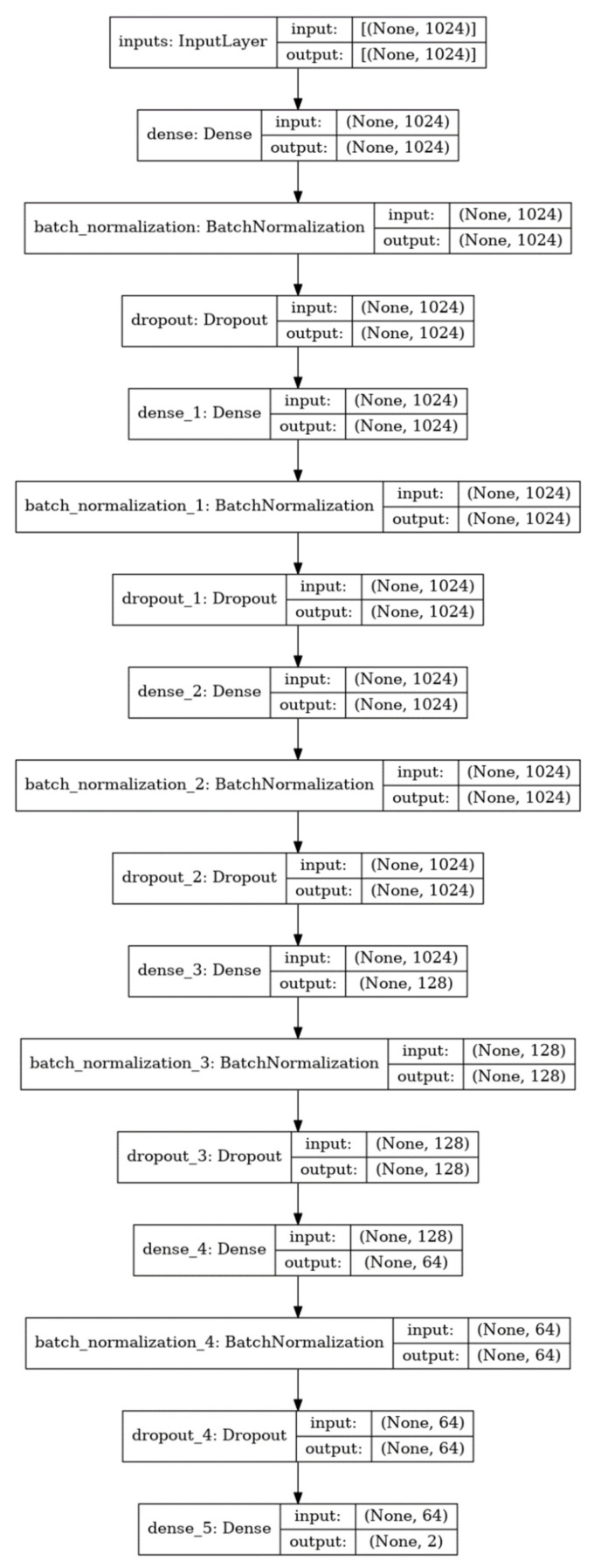
The deep neural network architecture of the DILI prediction model.

DNNs are fully connected networks consisting of input layers, hidden layers, and output layers [52]. Each layer has nodes that are connected to all nodes in the next layer with weights that can be trainable. Our model consisted of one input layer, five hidden layers, and one output layer. The input layer had 1024 nodes of the same length as the size of the ECFP4 fingerprint. The first, second, and third hidden layers had nodes of the same size as the input layer. The fourth and fifth hidden layers had 128 and 64 nodes, respectively. Each hidden layer had a batch normalization layer for stable training, a dropout layer with a ratio of 0.25 to limit overfitting, and a leaky rectified linear unit with a slope coefficient of 0.1, as the activation function. The output layer had two nodes corresponding to the one-hot encoded vectors of the DILI label (Figure 4).

Bernoulli NB, SVM, and RF models were implemented with the same training data sets to compare the performance of each model with that of the DNN model. For all ML methods, the Python scikit-learn package (https://www.scikit-learn.org/, version 0.24.2) was utilized to create each model. For the naive Bayes classification, the multivariate Bernoulli model was run with the BernoulliNB class from the package, where alpha was set to 1.0 as a smoothing parameter and fit_prior was set to “True” to allow the learning of class prior probabilities. The SVM model was created using the “SVC” class with the regularization parameter of 1.0, the kernel parameter of “rbf”, degree of polynomial kernel function as 3, and the gamma parameter of “scale” for the kernel coefficient. The RF model was constructed using the “RandomForestClassifier” class with the number of trees at 100, a max_depth of 10, and criteria of “gini” to designate the function of measurement of the quality of a split and other parameters to default.

### 4.3. Model Training

Model training, testing, and evaluation were performed using the Python package TensorFlow (version 2.5). At first, all weights in the DNN model were initialized with the He uniform initializer (https://www.tensorflow.org/api_docs/python/tf/keras/initializers/HeUniform) for better training. The Adam optimizer was used for weight optimization with a learning rate of 10^−5^. Categorical crossentropy was applied as the loss function for DILI classification. The number of epochs was set to 200, and the batch size was set to 64.

We implemented stratified 10-fold cross-validation for model training using the “StratifiedKFold” function of the Python scikit-learn library (https://www.scikit-learn.org/). With cross-validation, the training data set was divided into ten subsets while preserving the percentage of samples for each class; nine subsets were used to train the DNN model, while the remaining subset was used for validation. The procedure was repeated until every subset served as the test data set. In every round, if the loss of the test data set did not drop for ten consecutive epochs, the training process was forced to stop and a new training process was started. These cross-validation processes were repeated ten times, and the best trained model was selected based on the loss value of the test data sets. The best model was used for further studies, such as performance comparison, depending on molecular descriptors or model algorithms and evaluation of the validation data set and the external test data set.

### 4.4. Applicability Domain and Model Evaluation

We defined the applicability domain of the DNN model as the pool of substructures extracted from the training data set. Among the compounds from the validation data set or external data set, drugs that have at least one substructure absent from the pool can be classified as outliers. The substructures were represented as the integer ECFP4 bits that were calculated using the “GetMorganFingerprint“ function in the “AllChem” package of the RDKit library. We created a unique substructure pool from the entire training data set, and the substructures of each drug in the validation data set or the external test data set were compared with the pool to examine whether they were outliers.

The concept of endurance was introduced to test the robustness of the DNN model. It was defined as the ratio of substructures away from the applicability domain to all substructures of a drug. Different endurance levels were applied to evaluate the best DNN model with the validation data set or the external test data set. Six test subsets from the data set were prepared with endurance levels of 0%, 5%, 10%, 15%, 20%, or 30%, and the evaluation metrics for each test subset were calculated.

To further evaluate the performance of the DNN model, some DILI case-related drugs were collected from a literature survey using keywords such as “drug-induced liver toxicity” and “case reports”. From the search results, case reports from the last three years (since 2019) were chosen, and 12 new drugs, some already on the market and some that failed during clinical trials, were obtained. These drugs were tested for DILI risk to prove the utility of the DNN model in the real world.

### 4.5. Evaluation Metrics

All models were evaluated based on three metrics, namely, accuracy, sensitivity, and specificity, which were defined as follows:

(1) $$accuracy=\frac{\mathrm{TP}+\mathrm{TN}}{\mathrm{TP}+\mathrm{TN}+\mathrm{FP}+\mathrm{FN}}$$

(2) $$sensitivity=\frac{\mathrm{TP}}{\mathrm{TP}+\mathrm{FN}}$$

(3) $$specifity=\frac{\mathrm{TN}}{\mathrm{TN}+\mathrm{FP}}$$

(4) $$precision=\frac{\mathrm{TP}}{\mathrm{TP}+\mathrm{FP}}$$

(5) $$F1\;score=2\times \frac{precision\times sensitivity}{precision+sensitivity}$$

where TP, FP, TN, and FN represent true positive, false positive, true negative, and false negative, respectively. The F1 score and AUC values were calculated from the f1_score and AUC functions in the Python scikit-learn package, respectively.

## Figures and Tables

**Table 1:** Data sets used to generate the drug-induced liver injury (DILI)-prediction model in this study.

	Data Sets	DILI-Negatives	DILI-Positives	Total
Training data set	DILIrank data set	245	439	684
	LiverTox data set	234	22	256
	SUM	479	461	940
Validation data set	Greene data set	64	92	156
	Xu data set	10	13	23
	SUM	74	105	179

**Table 2:** Stratified 10-fold cross-validation results with ECFP4 over ten iterations.

Iteration	ACC	Best Loss
1	0.873 ± 0.0957	0.2271
2	0.864 ± 0.1074	0.1713
3	0.945 ± 0.0834	0.1264
4	0.918 ± 0.0725	0.1066
5	0.939 ± 0.0778	0.1276
**6**	**0.940 ± 0.0859**	**0.0837**
7	0.835 ± 0.1557	0.1858
8	0.927 ± 0.0788	0.1102
9	0.889 ± 0.0943	0.1258
10	0.814 ± 0.1928	0.2245

ACC: accuracy; DILI+: drug-induced liver injury (DILI)-positive; DILI−: DILI-negative. Bold represents the best model selected due to the lowest loss values.

**Table 3:** Comparison of the deep neural network models by molecular fingerprints.

**Endurance** **Level**	**ECFP4**				**ECFP6**			
	**ACC**	**SE**	**SP**	**F1**	**ACC**	**SE**	**SP**	**F1**
0%	0.731	0.714	0.750	0.741	0.750	0.500	1.000	0.667
5%	0.667	0.778	0.524	0.724	0.706	0.667	0.750	0.706
10%	0.648	0.744	0.500	0.719	0.615	0.667	0.556	0.651
15%	0.642	0.742	0.488	0.706	0.608	0.607	0.609	0.630
20%	0.632	0.763	0.434	0.706	0.571	0.571	0.571	0.593
30%	0.607	0.758	0.397	0.686	0.540	0.507	0.591	0.574
**Endurance** **Level**	**FCFP4**				**FCFP6**			
	**ACC**	**SE**	**SP**	**F1**	**ACC**	**SE**	**SP**	**F1**
0%	0.548	0.556	0.538	0.609	0.650	0.600	0.700	0.556
5%	0.553	0.635	0.452	0.630	0.618	0.632	0.600	0.611
10%	0.552	0.663	0.390	0.640	0.614	0.677	0.538	0.613
15%	0.531	0.638	0.379	0.622	0.586	0.640	0.513	0.626
20%	0.515	0.616	0.375	0.601	0.575	0.652	0.471	0.629
30%	0.517	0.606	0.392	0.600	0.551	0.629	0.449	0.604

ACC: accuracy, SE: sensitivity, SP: specificity, F1: F1 score.

**Table 4:** Evaluation results of the naive Bayesian, SVM, and RF models by endurance levels.

Endurance Level	Naive Bayesian				SVM				RF			
	ACC	SE	SP	F1	ACC	SE	SP	F1	ACC	SE	SP	F1
0%	0.538	0.643	0.417	0.600	0.577	0.571	0.583	0.593	0.577	0.500	0.667	0.560
5%	0.604	0.778	0.381	0.689	0.583	0.630	0.524	0.630	0.604	0.667	0.524	0.655
10%	0.606	0.767	0.357	0.702	0.592	0.651	0.500	0.659	0592	0.651	0.500	0.659
15%	0.606	0.803	0.302	0.711	0.615	0.667	0.535	0.677	0.578	0.652	0.465	0.652
20%	0.602	0.825	0.264	0.714	0.602	0.688	0.472	0.675	0.564	0.675	0.396	0.651
30%	0.589	0.810	0.279	0.697	0.601	0.684	0.485	0.667	0.571	0.674	0.426	0.646

ACC: accuracy; SE: sensitivity; SP: specificity; SVM: support vector machine; RF: random forest.

**Table 5:** Performance comparison of the DNN-based model with external data sets.

References	Level * (%)	Training Data Set Size	ACC	SE	SP	AUC
Liew et al. (entire data set) [12]	0%	114 (68+/46−)	0.789	0.838	0.717	0.853
	100%	187 (105+/82−)	0.642	0.724	0.537	0.742
valBLACK	0%	38	0.974	0.955	1.000	0.955
		(22+/16−)	(0.809)	(0.957)	(0.667)	(0.924)
	100%	47 (23+/24−)	0.830	0.957	0.708	0.937
valPAIR	0%	14	0.500	0.857	0.143	0.551
		(7+/7−)	(0.550)	(0.800)	(0.300)	(0.450)
	100%	20 (10+/10−)	0.450	0.700	0.200	0.525
valRANDOM	0%	62	0.742	0.769	0.696	0.836
		(39+/23−)	(0.750)	(0.819)	(0.646)	(0.595)
	100%	120 (72+/48−)	0.600	0.653	0.521	0.687
Zhang et al. [14]	0%	80	0.950	1.000	0.926	0.957
		(53+/27−)	(0.750)	(0.932)	(0.379)	(0.667)
	100%	85 (57+/28−)	0.941	0.982	0.857	0.952
Ai et al. [15]	0%	84	0.881	0.905	0.810	0.920
		(63+/21−)	(0.843)	(0.869)	(0.754)	(0.904)
	100%	121 (94+/27−)	0.893	0.904	0.852	0.911
Kotsampasakou et al. [13]	0%	151	0.636	0.595	0.687	0.672
		(84+/67−)	(0.600)	(0.670)	(0.520)	(0.642)
	100%	973 (524+/449−)	0.585	0.635	0.526	0.605

* Endurance level. ACC: accuracy; SE: sensitivity; SP: specificity; AUC: area under the receiver–operating characteristic curve. The data in parentheses are validation results from each reference.

**Table 6:** Evaluation results of the DNN model using 15 drugs with case reports.

Drugs	CID	Endurance Levels	Prediction	Prediction Probability
Flucloxacillin [26]	21,319	6.7%	DILI-positive	0.999
Aliskiren [27]	5,493,444	7.0%	DILI-positive	0.999
Rilpivirine [28]	6,451,164	5.0%	DILI-positive	0.994
Escitalopram [29]	146,570	5.0%	DILI-positive	0.989
Nilotinib [30]	644,241	7.6%	DILI-positive	0.982
Olmesartan [31]	158,781	6.3%	DILI-positive	0.974
Mesterolone [32]	15,020	4.1%	DILI-positive	0.971
Levothyroxine [33]	5819	3.9%	DILI-positive	0.965
Zanubrutinib [34]	135,565,884	6.4%	DILI-positive	0.922
Phenprobamate [35]	4770	2.8%	DILI-positive	0.896
Apixaban [36]	10,182,969	5.5%	DILI-positive	0.804
Fasiglifam [37]	24,857,286	7.0%	DILI-positive	0.660
Pirfenidone [38]	40,632	2.6%	DILI-positive	0.608
Ligandrol [39]	44,137,686	4.6%	DILI-negative	0.378
Ulipristal acetate [40]	130,904	6.5%	DILI-negative	0.036

DILI, drug-induced liver injury.

## Data Availability

All the data created in this study are deposited in Supplementary Materials and the datasets are available upon request.

## Supplementary Materials

The following data are available online, Table S1: List of substructures that existed only in the validation data set but not in the training data set; Table S2: Metrics of 10-fold stratified cross-validation over ten iterations; Table S3: Tanimoto distances between the same DILI classes; Table S4. The evaluation results on the 4 external data sets at all endurance levels.
