# Supplementary material for: Predictive Model for Drug-Induced Liver Injury Using Deep Neural Networks Based on Substructure Space
Source: Molecules. 2021 Dec 13;26(24):7548. doi: 10.3390/molecules26247548 (PMC8707960; doi:10.3390/molecules26247548)
Supplement: Supplementary file 1 [file molecules-26-07548-s001.zip › molecules-1512828-supplementary.pdf]

Table S1. List of substructures that existed only in validation dataset but not in training dataset.

Integer Bits	Bit SMILES	Compounds	Count
2282149209	CC(N)C(=O)[O-]	Azlocillin Sodium, Cloxacillin Sodium, Mezlocillin Sodium, Penicillin G Sodium, Dicloxacillin Sodium, Methicillin Sodium	6
2991692856	CN(C)C(C(=O)[O-])C(C)(C)S	Azlocillin Sodium, Cloxacillin Sodium, Mezlocillin Sodium, Penicillin G Sodium, Dicloxacillin Sodium, Methicillin Sodium	6
2933418651	ccn(cn)CC	Econazole Nitrate, Butoconazole Nitrate, Miconazole, Sulconazole Nitrate	4
2142685628	CNCCN	gatifloxacin, Temafloxacin, Lomefloxacin HCl, grepafloxacin	4
1507955614	CCNC(C)C	gatifloxacin, Temafloxacin, Lomefloxacin HCl, grepafloxacin	4
1260363809	CC(N)CN(c)C, CC(N)CN(C)c	gatifloxacin, Temafloxacin, Lomefloxacin HCl, grepafloxacin	4
1040797185	CNC(C)CN	gatifloxacin, Temafloxacin, Lomefloxacin HCl, grepafloxacin	4
2017861928	CC(C)N	gatifloxacin, Temafloxacin, Lomefloxacin HCl, grepafloxacin	4
2794204746	cccc(c)[N+]	Nicardipine HCl, 1,3-dinitrobenzene, Nimodipine	3
4264704113	CCl	Beclomethasone Dipropionate, Mometasone Furoate, Ciprofibrate	3
2871146062	CC(C)(N)O	Cefoxitin, Cefotetan, Moxalactam Disodium	3
618008133	CC(C)(c)c, CC(C)(c)C	Glutethimide, Aminoglutethimide, Maprotiline	3
3003276483	CN1CC(N)(O)C1=O	Cefoxitin, Cefotetan, Moxalactam Disodium	3
250814365	ccc(Cl)c(c)C	Econazole Nitrate, Miconazole, Sulconazole Nitrate	3
3526465279	CC(C)(C)C	Beclomethasone Dipropionate, Mometasone Furoate, Dexamethasone	3
3322213908	CC(C)CC(C)C	Beclomethasone Dipropionate, Mometasone Furoate, Dexamethasone	3
3197242792	CCC(C(C)C)C(C)(C)C	Beclomethasone Dipropionate, Mometasone Furoate, Dexamethasone	3
1974156900	CC(=O)NC(C)=O	Glutethimide, Aminoglutethimide, Ethosuximide	3
821753002	CC=CC(C)(C)C	Beclomethasone Dipropionate, Mometasone Furoate, Dexamethasone	3
883850840	CC=C(CC)C(C)(C)C	Beclomethasone Dipropionate, Mometasone Furoate, Dexamethasone	3
2132058917	cccc(c)C	Econazole Nitrate, Miconazole, Sulconazole Nitrate	3
393605944	COC(C)(C)N	Cefoxitin, Cefotetan, Moxalactam Disodium	3
3749319234	cc(c)N(CC)CC	clinafloxacin, gatifloxacin, Lomefloxacin HCl	3
707917694	ccc(Cl)c(c)O	Bithionol, Hexachlorophene	2
3360753543	ccc(cc)N(C)N	Phenylbutazone, Sulfinpyrazone	2
451273429	cN(C)N, CN(c)N	Phenylbutazone, Sulfinpyrazone	2
1725252687	cc(c)N(C(C)=O)N(c)C, cc(c)N(C(C)=O)N(C)c	Phenylbutazone, Sulfinpyrazone	2

2584059174	CC(C)C(=O)N(c)N	Phenylbutazone, Sulfinpyrazone	2
1044405971	ccc(Cl)cc	3,5-Dichloroaniline hydrochloride, Bithionol	2
1719784028	ccc(nc)N(C)C	Tripelennamine HCl, Methapyrilene	2
3311951855	CN(c)CCN	Tripelennamine HCl, Methapyrilene	2
2688692504	cc(c)CN(C)c	Tripelennamine HCl, Bepridil HCl	2
3565565663	cc(n)N(CC)Cc	Tripelennamine HCl, Methapyrilene	2
3675674571	cc(c)N(CC)CC	Temafloxacin, grepafloxacin	2
1099870031	CCOC(C)=O	Azaserine, Octyl Methoxycinnamate	2
4020206018	CC(C)C(Cl)(C(C)O)C(C)(C)C	Beclomethasone Dipropionate, Mometasone Furoate	2
3835029422	CCC(C(C)C)C(C)(C)Cl	Beclomethasone Dipropionate, Mometasone Furoate	2
2008452727	CCC(O)C(C)(C)Cl	Beclomethasone Dipropionate, Mometasone Furoate	2
1887419926	CCC(C)(C(C)C)C(C)(C)O	Beclomethasone Dipropionate, Mometasone Furoate	2
1299092894	CCC(C)C(C)(C)O	Beclomethasone Dipropionate, Mometasone Furoate	2
3278383012	COC(OC)C(C)N	Gentamycin, Tobramycin	2
2417042713	ccc(c(c)S)N(C)c	AY-25329, Moricizine HCl	2
1314885574	CC(N)C(c)O	Phenylpropanolamine HCl, Methoxamine HCl	2
982863348	CC(C)CC(C)O	Chenodiol, Lithocholic acid	2
4236127874	CCCC(C)(c)C	Glutethimide, Aminoglutethimide	2
2961123579	ccc(cc)C(C)(C)C	Glutethimide, Aminoglutethimide	2
2512391063	cc(c)C(CC)(CC)C(N)=O	Glutethimide, Aminoglutethimide	2
933123185	CNC(=O)C(C)(C)c	Glutethimide, Aminoglutethimide	2
4143286053	cOC(C)(C)C(=O)O	Bezafibrate, Ciprofibrate	2
3234389540	cc(s)CC(N)=O	Cephalothin Sodium, Cefoxitin	2
887206553	CCC(C)(c)C	Glutethimide, Aminoglutethimide	2
347621640	CSC1N(C)CC1(N)O	Cefoxitin, Cefotetan	2
618638902	CNC1(OC)C(=O)NC1S	Cefoxitin, Cefotetan	2
188636353	ccc(CC)c(c)C, ccc(Cc)c(c)-c	Ketotifen, 2-acetylaminofluorene (2-AAF)	2
1293522643	C=C(C)N1C(=O)CC1S	Cefoxitin, Cefotetan	2
2508216837	CCSC(C)N	Cefoxitin, Cefotetan	2
2946958508	CC(N)S	Cefoxitin, Cefotetan	2
3839181794	NCCC(N)=O	AY-25329, Moricizine HCl	2
2523730310	ccc(c(c)S)N(C)c, ccc(c(c)S)N(c)C	AY-25329, Moricizine HCl	2
4252229081	CC(C)N	Phenylpropanolamine HCl, Methoxamine HCl	2
1413351488	cc(c)N(C(C)=O)c(c)c	AY-25329, Moricizine HCl	2
199116476	CCC(=O)N(c)c	AY-25329, Moricizine HCl	2
2727439157	CCC(C(N)=O)C(N)=O	Phenylbutazone, Sulfinpyrazone	2
1128360814	C=C(C)C(C(=C)C)c(c)c	Nicardipine HCl, Nimodipine	2
2226908007	ccc(cc)C(C)C	Nicardipine HCl, Nimodipine	2
3400020746	cCOC(C)c	Econazole Nitrate, Miconazole	2
3498777222	cc(c)CN(C)C	Pargyline, Nicardipine HCl	2

1094126528	ccc(c(c)Cl)C(C)O	Econazole Nitrate, Miconazole	2
714883344	cn(c)CC(c)O	Econazole Nitrate, Miconazole	2
283306526	cc(c)C(Cn)OC	Econazole Nitrate, Miconazole	2
97722113	cc(=O)oc(c)c	Novobiocin, aflatoxin B1	2
22113891	CC(C)(C)O	Beclomethasone Dipropionate, Mometasone Furoate	2
973747042	COC(C(C)=O)(C(C)C)C(C)(C)C	Beclomethasone Dipropionate, Mometasone Furoate	2
286827626	C=CC(C)(C(=C)C)C(C)(C)Cl	Beclomethasone Dipropionate, Mometasone Furoate	2
532959195	cc(C)cc(c)[N+]	Nicardipine HCl, Nimodipine	2
1082128421	CC(C)(C)Cl	Beclomethasone Dipropionate, Mometasone Furoate	2
3871470382	CC(Cl)C(Cl)C(C)Cl	1,2,3,4,5,6-hexachlorocyclohexane	1
2128737333	CC(C)Cl	1,2,3,4,5,6-hexachlorocyclohexane	1
4265486248	CCl	1,2,3,4,5,6-hexachlorocyclohexane	1
996966525	O=[Cr]	potassium dichromate	1
1646253800	ccc(c(c)O)C(C)(C)C	Probucol	1
525112373	cc(C)cc(c)S	Probucol	1
3621277923	C[N+](C)(C)CCO	Gallamine Triethiodide	1
3059039003	CC[N+](CC)(CC)CC	Gallamine Triethiodide	1
1130676539	cOCC[N+]	Gallamine Triethiodide	1
1035126318	CCCC(C)(C)C	Carbenoxolone Disodium	1
3316519268	ccc(OC)c(c)C	Methicillin Sodium	1
4081537576	cc(-c)ccn	Amrinone	1
526788212	CCCN(C)C	Demecarium Bromide	1
2487278030	ccc(Nc)c(c)S	phenothiazine	1
1234114758	ccc(Sc)c(c)N	phenothiazine	1
210610014	O=[Cr](=O)([O-])O[Cr]	potassium dichromate	1
2491708295	CCCN(C)C	Bepridil HCl	1
3788220532	ccc(c(c)O)N(C)c	ragaglitazar	1
997159370	[O-][Cr]	potassium dichromate	1
360574553	ccc(Oc)c(c)N	ragaglitazar	1
3840912935		potassium dichromate	1
712583196	cO[Bi], CO[Bi]	Bismuth Subsalicylate	1
1521777139	ccc(cc)OC	Bambuterol	1
1040949262	cc(C)c(O)c(c)C	Probucol	1
960859226	ccc(cc)[N+](C)(C)C	Demecarium Bromide	1
1614118283	CCN(C)C(=O)O	Demecarium Bromide	1
3610518552	cc([N+])cc(c)O	Demecarium Bromide	1
4283171114	cC(=O)NC(C)C	Iopamidol	1
4231393221	cc(c)C(=O)NC	Iopamidol	1
4197496443	CNC(CO)CO	Iopamidol	1
1089669812	cc(S)ccn	Cephapirin Sodium	1

708322256	CC[N+](C)(C)C	Oxyphenonium	1
2293665300	ccc(cc)SC	Probucol	1
1608637670	cSC, CSc	Probucol	1
4249144910	O=[Cr](=O)([O-])O	potassium dichromate	1
4119428047	cc(c)SC(C)(C)S	Probucol	1
3363980958	CC=CC(=O)O	maleic acid	1
1391877500	cc(S)cc(c)Cl	Bithionol	1
2776666305	ccc(Sc)c(c)O	Bithionol	1
3972042419	cc(N)cc(c)Cl	3,5-Dichloroaniline hydrochloride	1
2434661383	ccc(c(c)C)C(C)(C)c	Maprotiline	1
144561080	ccc(Cl)c(c)S	Butoconazole Nitrate	1
374795611	ccc(c(c)C)C(C)c	Maprotiline	1
4219643190	CC(C)CN(C)N	Gliclazide	1
605166804	cc(S)c(O)c(c)Cl	Bithionol	1
2773314963	Cl[Cd]	cadmium chloride	1
4023220930	CNC(=O)C(C)(O)O	Alloxan Hydrate	1
3097783358	cccc(c)N	Phenazopyridine HCl	1
1216233818	cc(O)c(Cc)c(c)Cl	Hexachlorophene	1
1333269610	cccc(c)S	Sulfinpyrazone	1
3724702181	cccc(c)Br	Bromobenzene	1
1418166502	cc(C)c(Cl)c(c)Cl	Hexachlorophene	1
1890008402	cc(Cl)cc(c)Cl	Hexachlorophene	1
1808277211	CCCC(C)(C)C	Ciglitizone	1
364062151	cc(C)c(O)c(c)Cl	Hexachlorophene	1
1471429178	NS(=O)(=O)O	Aztreonam E-isomer	1
1848279521	CC1CC(=O)N1S(=O)(=O)O	Aztreonam E-isomer	1
43831371	CNC1C(=O)NC1C	Aztreonam E-isomer	1
891256307	cncc(C)n	Pilocarpine	1
500158515	CC(N)C(O)C(C)N	Spectinomycin HCl	1
1532304569	CCC(OC)C(C)(C)C	estradiol 17b glucuronide	1
3433697626	cc(C)n(O)c(c)=O	Ciclopirox	1
2618939437	ccc(C(C)C)n(c)O	Ciclopirox	1
1121245966	cc(C)cc(n)=O	Ciclopirox	1
452370461	COC(=O)C(C)C	Pilocarpine	1
2780825838	c[nH]c(CC)c(c)C	Molindone HCl	1
276362736	COCC(C)C	Pilocarpine	1
4259783135	CC(C)CN(C)C	Molindone HCl	1
3662324687	cc(C)c(CC)c(C)[nH]	Molindone HCl	1
142293962	COC(OC)C(C)(O)O	Spectinomycin HCl	1
2388124599	NC1CN(S)C1=O	Aztreonam E-isomer	1
2662121690	CC1C(N)CN1S	Aztreonam E-isomer	1
3920733342	CN(C)S(=O)(=O)O	Aztreonam E-isomer	1
4276481664	CN(C)S	Aztreonam E-isomer	1

1195341853	cCC(CO)C(C)C	Pilocarpine	1
217636340	CC(C)OC(C)O	Spectinomycin HCl	1
442135962	cc(C)[nH]c(c)C	Molindone HCl	1
48608183	CCC(=O)C(C)(O)O	Spectinomycin HCl	1
3207543589	cc(C)c(C(C)=O)c(C)[nH]	Molindone HCl	1
910540935	cncn(c)C	Pilocarpine	1
3708206101	CCC(C(=O)O)C(C)C	Pilocarpine	1
1249523921	cn(C)c(cn)CC	Pilocarpine	1
1355914862	ccn(CC)c(c)n	Nalidixic Acid	1
797709143	ccc(NC)c(c)c	Hycanthone	1
893123477	cc(c)c(=O)c(c)c	Hycanthone	1
989264549	ccc(CO)c(c)s	Hycanthone	1
1719116140	csc(c(c)C)c(c)c	Hycanthone	1
2162253520	ccc(sc)c(c)c	Hycanthone	1
2778456986	cc(c)sc(c)c	Hycanthone	1
3848223426	cc(N)c(c(c)=O)c(c)s	Hycanthone	1
1528282272	ccc(C)on	Isoxicam	1
957070648	ccc(=O)n(c)O	Ciclopirox	1
1402077519	cccc(C)n	Nalidixic Acid	1
1962060547	cc(C)nc(c)n	Nalidixic Acid	1
2230330662	ccc(c(c)=O)c(n)n	Nalidixic Acid	1
3544348331	cnc(c(c)c)n(c)C	Nalidixic Acid	1
1400777067	ccc(no)NC	Isoxicam	1
4059302282	ccc(C)nc	Nalidixic Acid	1
805987832	Cc(o)cc(n)N	Isoxicam	1
186700734	cNCCN	Hycanthone	1
11046599	ccc(c(c)=O)c(c)s	Hycanthone	1
3034137634	CCN(CC)C(N)=O	Diethylcarbamazine	1
2076122780	CCN(CC)C(N)=O	Diethylcarbamazine	1
1703453026	CCC(C)C	Pilocarpine	1
1951940009	cc(n)CC(C)C	Pilocarpine	1
407520881	cc(C)cc(C)n	Ciclopirox	1
192187741	cc(n)C(CC)CC	Ciclopirox	1
2340118693	cc(C)n(C)cn	Pilocarpine	1
1406989977	COC(C(C)O)C(C)O	Spectinomycin HCl	1
1051813428	C=CC(=O)OC	Octyl Methoxycinnamate	1
3661262542	cc(C)o	Isoxicam	1
2231359664	cC=CC(=O)O	Octyl Methoxycinnamate	1
106928295	CCCCC	Phenylbutazone	1
1100883889	CNC(CO)C(c)O	Chloramphenicol Palmitate	1
2895999632	conc(c)N	Isoxicam	1
3501351633	COCC(C)N	Chloramphenicol Palmitate	1
1660358100	CN(C)C(=O)N(C)C	Diethylcarbamazine	1

1805166875	NC(N)=O	Diethylcarbamazine	1
643424422	CC(N)C(O)C(C)O	Spectinomycin HCl	1
2719762529	CC(C)(C)C	Ethosuximide	1
2399703789	CCC(C)OC	Spectinomycin HCl	1
2059488795	ccc(c(c)F)N(C)C	grepafloxacin	1
9713885	cc(C)c(c(c)=O)c(c)n	grepafloxacin	1
119562847	cc(C)c(F)c(c)N	grepafloxacin	1
2730755516	CCC(C)C(C)O	Troleandomycin	1
2729628705	cc(N)c(F)c(c)n	Lomefloxacin HCl	1
1642318146	cc(C)c(=O)c(c)c	grepafloxacin	1
2497108876	COC(C(C)N)C(O)O	Troleandomycin	1
2771442675	ccc(c(c)c)n(c)C	grepafloxacin	1
3349573461	CNC(C(C)O)C(C)O	Spectinomycin HCl	1
3745247955	cc(F)c(C)c(c)c	grepafloxacin	1
1418595958	ccn(-c(c)c)c(c)c	Temafloxacin	1
4208723524	ccc(c(c)c)n(c)-c	Temafloxacin	1
5559840	cNC(C)N(C)c	Metolazone	1
1688867222	CC(N)N	Metolazone	1
1956113409	cc(c)N(C(c)=O)C(C)N	Metolazone	1
4057785929	CCC(C)C(C)(C)O	Dexamethasone	1
3608122364	CCC(C)(C(C)C)C(C)(C)O	Dexamethasone	1
2433422477	CC(C)(C)O	Dexamethasone	1
2098686412	C=CC(C)(C(=C)C)C(C)(C)F	Dexamethasone	1
1950936949	CC(=O)C(O)(C(C)C)C(C)(C)C	Dexamethasone	1
1588425244	CC(C)(O)C(=O)CO	Dexamethasone	1
14114542	CC(C)C(F)(C(C)O)C(C)(C)C	Dexamethasone	1
4137181360	cc(o)C(=O)OC	Mometasone Furoate	1
4097606248	CC(C)(O)C(=O)CCl	Mometasone Furoate	1
2812104872	CC(=O)C(C)C(C)O	Troleandomycin	1
3005152558	CC(C)CC(C)(C)O	Troleandomycin	1
3022110153	CCC(C(C)O)N(C)C	Troleandomycin	1
3496030858	CC(O)C(C)C(C)O	Troleandomycin	1
2932363931	CCCl	Mometasone Furoate	1
136402006	cC(=O)OC(C)(C)C	Mometasone Furoate	1
1479119891	ccc(oc)C(=O)O	Mometasone Furoate	1
1370911154	CC(=O)CCl	Mometasone Furoate	1
2394757384	COC(C(C)C)C(C)C	Troleandomycin	1
2021476951	ccc(C)c(c)N	Metolazone	1
2188426911	ccc(NC)c(c)C	Metolazone	1
2745711692	CNC(=O)N(C)C	Mephobarbital	1
3411698582	COC(C(C)N)C(C)O	Spectinomycin HCl	1
3590415191	COC(O)(C(C)=O)C(O)O	Spectinomycin HCl	1
3605235311	CC(=O)CC(C)O	Spectinomycin HCl	1

3823995908	CNC(C(C)O)C(C)O	Spectinomycin HCl	1
2786802479	CCC(=O)NC	Ethosuximide	1
4036320547	CC(C)OC(C)O	Spectinomycin HCl	1
4122325276	CC(O)O	Spectinomycin HCl	1
136353966	COC(C(C)C)C(C)C	Troleandomycin	1
274384155	COC(C(C)O)C(C)O	Troleandomycin	1
463959832	CC(C)C(=O)C(C)(C)O	Troleandomycin	1
2568538409	CNC(=O)C(C)(C)C	Ethosuximide	1
1860296569	CC(C)(C)CC(N)=O	Ethosuximide	1
693166148	CCC(C)(C)C	Ethosuximide	1
249787322	CCC(C)(CC)C(N)=O	Ethosuximide	1
2539999501	cc(c)C(CC)(C(N)=O)C(N)=O	Mephobarbital	1
2533335663	ccc(c(c)C)N(C)C	Metolazone	1
2136193252	CC(=O)NC(N)=O	Mephobarbital	1
1809978325	CC(=O)N(C)C(N)=O	Mephobarbital	1
608763756	CN(C)C(=O)C(C)(C)c	Mephobarbital	1
4172736314	CCC(=O)OC	Beclomethasone Dipropionate	1
3411404463	CCC(=O)OC	Beclomethasone Dipropionate	1
586976137	CCC1(C(C)=O)CO1	Troleandomycin	1
622233516	CC(O)C(C)C(C)O	Troleandomycin	1
2211217220	CC(C)(C)O	Troleandomycin	1
3115087023	CC(=O)OC(C)(C)C	Beclomethasone Dipropionate	1
1451402951	CC(C)(O)C(=O)CO	Beclomethasone Dipropionate	1
2373710881	CC1(C)CO1	Troleandomycin	1
4032994621	cc(c)NC(C)N	Metolazone	1
3478223599	cc(C)cc(c)S	Metolazone	1
2887626532	ccc(C(N)=O)c(c)N	Metolazone	1
1477785326	cc(F)c(c(c)F)N(C)C	Lomefloxacin HCl	1
26756162	O=S(=O)([O-])[O-]	FeSO4	1
3480856402	ccn(CC)c(c)c	Lomefloxacin HCl	1
2587571567	cc(c)CCC	tacrine	1
2726983370	C=NOCC	fluvoxamine	1
4111903471	CON=C(C)c	fluvoxamine	1
1404983490	[Cr]O[Cr]	potassium dichromate	1
3071342759	O=[Cr](=O)([O-])O[Cr](=O)(=O)[O-]	potassium dichromate	1
440938400	CC=[N+]	Azaserine	1
1143583768	CC=[N+]=[N-]	Azaserine	1
1528365532	[N-]=[N+]=CC(=O)O	Azaserine	1
2739538070	C=[N+]=[N-]	Azaserine	1
3510037322	COC(=O)C=[N+]	Azaserine	1
3906444363	NC(CO)C(=O)O	Azaserine	1
3973036443	COCC(C)N	Azaserine	1
1388553841	COC(O)C(C)N	d-galactosamine	1

2745868662	CC(O)C(N)C(O)O	d-galactosamine	1
1425147615	cc(F)c(c(c)Cl)N(C)C	clinafloxacin	1
1692051488	CC(N)CCN	clinafloxacin	1
2346104032	cc(N)c(Cl)c(c)n	clinafloxacin	1
3350675456	cc(Cl)c(c(c)c)n(c)C	clinafloxacin	1
101365395	cc(C)c(N)c(c)c	tacrine	1
252721428	cnc(CC)c(c)C	tacrine	1
2260741221	CCCC(c)=N	fluvoxamine	1
1926549088	CC(c)=N	fluvoxamine	1
1768844655	ccc(cc)C(C)=N	fluvoxamine	1
3591429776	C=CC(c(c)c)C(O)O	aflatoxin B1	1
2138253685	cc(C)c(C(C)=O)c(=O)o	aflatoxin B1	1
2396650387	CC=COC	aflatoxin B1	1
2423454930	coc(c(c)C)c(c)c	aflatoxin B1	1
2693552502	COC(Oc)C(c)C	aflatoxin B1	1
2698958207	COC	aflatoxin B1	1
3381816528	cc(O)c(c(c)o)C(C)C	aflatoxin B1	1
3410570877	cc(C)c(c(c)O)c(c)o	aflatoxin B1	1
3517255556	cc(c)C(=O)CC	aflatoxin B1	1
4249405884	cc(C)c(CC)c(c)c	aflatoxin B1	1
1754393348	cc(c)C(CC)=NO	fluvoxamine	1
264762917	CC(C)Nc(n)n	Astemizole	1
548571000	ccc(c(c)n)n(c)C	Astemizole	1
833123915	cc(c)nc(n)N	Astemizole	1
1391940372	cc(c)n(Cc)c(n)N	Astemizole	1
2506328221	nc(n)N	Astemizole	1
3408765322	cnc(NC)n(c)C	Astemizole	1
921629099	CON	fluvoxamine	1
1733160153	NCCON	fluvoxamine	1
832239758	cc(N)c(CC)c(C)n	tacrine	1
3204810638	ccc(c(c)N)c(c)n	tacrine	1
1535536404	cCCC(c)=O	aflatoxin B1	1
3902071045	cc(N)cc(c)O	3-Acetamidophenol	1
2097164133	CC(N)C(O)C(n)O	puromycin	1
2320270130	CNC(C(C)O)C(C)O	puromycin	1
3552912573	cc(n)N(C)C	puromycin	1
3867221932	cnc(c(c)n)N(C)C	puromycin	1
4142489433	cnc(c(n)N)c(n)n	puromycin	1
4195181160	COC(CO)C(C)N	puromycin	1
56662293	C=CCCN	Thiothixene	1
2126686894	cc(c)S(=O)(=O)N(C)C	Thiothixene	1
2486110993	ccc(cc)S(N)(=O)=O	Thiothixene	1
3169904928	C=C(c)c(cc)c(c)S	Thiothixene	1

3194811565	C=C(c)c(cc)c(c)S	Thiothixene	1
3894544496	CN(C)S(c)(=O)=O	Thiothixene	1
4069016315	CN(C)S	Thiothixene	1
8757308	CCCC(C)(c)c	Maprotiline	1
778085759	CCCC(C)(c)c	Maprotiline	1
1336346143	cc(c)C(CC)c(c)c	Maprotiline	1
3648820092	CCCCN	Maprotiline	1
3747360616	CCCC(c)c	Maprotiline	1
3801831583	cc(c)C(CC)(CC)c(c)c	Maprotiline	1
2712323852	Cl[Cd]Cl	cadmium chloride	1
405987842		cadmium chloride	1
3635585100	CCCOc	oxybendazole	1
1552769110	CC(C)(O)C(C)(O)C(=O)O	Monocrotaline	1
263110429	CC(N)=S	thioacetamide	1
1781691773	CCN(CC)NC	Gliclazide	1
236703256	CC(C(=O)O)C(C)(C)O	Monocrotaline	1
293133348	CC(C)=CCN	Monocrotaline	1
1137171426	CCC(OC)C(C)N	Monocrotaline	1
1166572987	COC(=O)C(C)(C)O	Monocrotaline	1
1288540776	CC(O)CCN	Monocrotaline	1
1439835860	CC(=O)OC(C)C	Monocrotaline	1
1931975494	COC(=O)C(C)C	Monocrotaline	1
2636383078	CCCO	oxybendazole	1
1960278286	C=CCN(C)C	Monocrotaline	1
2038424846	CC(C)(C)O	Monocrotaline	1
2456970889	CC(C)C	Monocrotaline	1
2766857770	C=C(C)C(C(C)O)N(C)C	Monocrotaline	1
3193649093	CC(C)C(C)(O)C(C)(C)O	Monocrotaline	1
3219202921	CC=C(CO)C(C)N	Monocrotaline	1
3678038791	CCN(CC)C(C)C	Monocrotaline	1
3788138975	CC(C)(C)O	Monocrotaline	1
1664970050	cc(O)cc(c)O	aflatoxin B1	1
873875243	C=COC(C)O	aflatoxin B1	1
3678650707	cc(F)c(c(c)c)n(c)C	Lomefloxacin HCl	1
938376246	CC(=O)NC(C)(C)O	Moxalactam Disodium	1
2895211994	CC(=O)C(=C(c)O)C(C)C	Methacycline HCl	1
114560468	C=C(O)c(c(c)C)c(c)O	Demeclocycline HCl	1
149714485	CC(=O)C(=C(c)O)C(C)C	Demeclocycline HCl	1
579427249	cc(C)c(c(c)Cl)C(C)O	Demeclocycline HCl	1
1533716076	C=C(C)C(CC)C(c)O	Demeclocycline HCl	1
2012497532	ccc(Cl)c(c)C	Demeclocycline HCl	1
3166013939	cc(c)C(O)C(C)C	Demeclocycline HCl	1
32590473	ccc(CN)sc	Methapyrilene	1

3064055020	cc(s)CN(C)c	Methapyrilene	1
990675664	CCn(nn)c(n)S	Cefotiam HCl	1
2313223640	CSc(nn)n(C)n	Cefotiam HCl	1
4275705722	cc(c)cc(c)c	3-Methylcholanthrene	1
2478873394	cn(n)CCN	Cefotiam HCl	1
2891647640	cn(C)nnn	Cefotiam HCl	1
767854107	ccc(cc)C(N)=O	Trimethobenzamide HCl	1
1813252713	C#CCN(C)C	Pargyline	1
1972843467	CCN(C)Cc	Pargyline	1
145061073	CNC(=O)C(c)C	Moxalactam Disodium	1
602625333	CC(c)C(=O)[O-]	Moxalactam Disodium	1
1917154713	C=C(C)c(cc)c(c)C	Methacycline HCl	1
1828093636	C=C(O)c(c(c)C)c(c)O	Methacycline HCl	1
1442088011	C=C(c)C(C(=C)C)C(C)O	Methacycline HCl	1
211882074	CCCSC	ethionine	1
3975907156	cc(N)cc(c)N	Moricizine HCl	1
425258879	cN(c)CCO	ragaglitazar	1
1400619195	CCOC(C)C	ragaglitazar	1
2951019787	cCC(OC)C(=O)O	ragaglitazar	1
3734616521	CC(O)C(=O)O	ragaglitazar	1
3917675302	CC(C)O	ragaglitazar	1
4051892429	cc(c)CC(C)O	ragaglitazar	1
804536389	cc(c)OCC	Phenacetin	1
631714732	CCSC	ethionine	1
181560361	C=C(c(c)c)C(C)C	Methacycline HCl	1
1791942650	CCSCC	ethionine	1
4095742117	CCS	ethionine	1
190764063	CNC(=O)N(C)N	Streptozocin	1
1103347330	CN(C)N=O	Streptozocin	1
1552685013	CN(N=O)C(N)=O	Streptozocin	1
2924668923	CC(C)NC(N)=O	Streptozocin	1
3873245682	CN(C)N	Streptozocin	1
1264681	CC(C)C(O)C(C)C	Methacycline HCl	1
869414681	CC(N)=C(CO)CS	Moxalactam Disodium	1
1106437574	C=C(C)N1C(=O)CC1O	Moxalactam Disodium	1
852197660	cc(c)OC(C)O	aflatoxin B1	1
1187709100	C=C(C)COC	Moxalactam Disodium	1
4182551454	cc(c)N(C)S(c)(=O)=O	tianeptine	1
2358246356	C=C(c)c(cc)c(C)s	Ketotifen	1
3283736102	cc(C)ccs	Ketotifen	1
3365255225	cc(s)C(=O)Cc	Ketotifen	1
3595322842	ccsc(c)C	Ketotifen	1
3918283204	csc(C(C)=O)c(c)C	Ketotifen	1

3776148706	cc(C)cc(c)N	trifluoperazine	1
313683681	ccc(c(c)C)N(C)C	mianserin	1
3696580341	cc(c)N(CC)C(C)c	mianserin	1
97126450	cCCNC	Betahistine DiHCl	1
3031806053	cc(n)CCN	Betahistine DiHCl	1
225605541	cc(c)C(=O)c(c)s	Nocodazole	1
1261901580	cCC(=O)NC	Cefoxitin	1
2111809098	CC(=O)NC(C)(C)O	Cefoxitin	1
1705430016	cc(c)c(Nc)c(c)c	Amsacrine HCl	1
17206499	ccc(OC)c(c)c	aflatoxin B1	1
145622424	cC(C)C=CO	aflatoxin B1	1
542597728	ccc(OC)c(c)C	aflatoxin B1	1
821651104	coc(=O)c(c)C	aflatoxin B1	1
3852990270	ccc(c(c)N)C(c)N	tianeptine	1
2263178789	ccc(c(c)S)C(c)N	tianeptine	1
1621243199	ccc(c(c)C)S(N)(=O)=O	tianeptine	1
729796570	C=C1SC(C)S1	Cefotetan	1
1218968670	CCOC(C)N	Moxalactam Disodium	1
1765956250	CNC1(OC)C(=O)NC1O	Moxalactam Disodium	1
2258459237	COC1N(C)CC1(N)O	Moxalactam Disodium	1
2981901713	cc(c)C(C(N)=O)C(=O)[O-]	Moxalactam Disodium	1
155917746	C=C(C)C(N)=O	Cefotetan	1
242039070	CC(C)=C1SCS1	Cefotetan	1
390092133	CC(S)S	Cefotetan	1
473778896	C=C(C)C	Cefotetan	1
1436597828	C=C(C)C(=O)O	Cefotetan	1
1253603500	cc(S)cc(c)Cl	tianeptine	1
2586096179	NC(=O)C(C(=O)O)=C(S)S	Cefotetan	1
3122152552	CC(=O)NC(C)(C)O	Cefotetan	1
3460870405	C=C(S)S	Cefotetan	1
3554910938	NC(=O)C1SCS1	Cefotetan	1
4145588818	CNC(=O)C(S)S	Cefotetan	1
765502559	cc(c)C(O)C(N)=O	Cefamandole Sodium	1
4185376836	CNC(=O)C(c)O	Cefamandole Sodium	1
239709072	ccc(c(c)C)N(C)S	tianeptine	1
503074166	cnoc(c)C	Isoxicam	1
1757369339	COC(C(C)O)C(C)(C)O	Novobiocin	1
4245637465	ccc(c(c)c)c(c)c	3-Methylcholanthrene	1
1428527756	NCN	Fluspirilene	1
3572254755	CON	acivicin	1
3728045287	C=NOC(C)C	acivicin	1
825301973	CC(N)CN(C)C	AY-25329	1
947260286	CCC(CN)N(C)C	AY-25329	1

451148100	CCN(CC)C(C)=O	Praziquantel	1
1957108180	cC(N)CN(C)C	Praziquantel	1
2850339349	CN(C)C(=O)CN	Praziquantel	1
4083739724	CN(C)CC(N)=O	Praziquantel	1
4212533435	cCCN(C)C	Praziquantel	1
1716880071	CC(=O)NCN	Fluspirilene	1
681840918	cc(c)nc(C)[nH]	Mibefradil	1
1732858705	CNC(=O)C(C)(C)N	Fluspirilene	1
1926294990	CCC(CC)(C(N)=O)N(C)c	Fluspirilene	1
2230886913	cc(c)N(CN)C(C)(C)C	Fluspirilene	1
2716676786	CNCN(c)C	Fluspirilene	1
3863742748	ccc(cc)N(C)C	Fluspirilene	1
1863936778	CCOC(N)=O	Furazolidone	1
2359942291	COC(=O)N(C)N	Furazolidone	1
3423490106	CN(N)CCO	Furazolidone	1
3729757522	COCCN	Furazolidone	1
3460934208	C=NO	acivicin	1
3314869458	CC(N)C(=O)O	acivicin	1
3296500885	CC(=N)Cl	acivicin	1
3022144085	CCC(Cl)=NO	acivicin	1
3049074880	ncc(F)c([nH])N	Flucytosine	1
3362390064	ccnc([nH])=O	Flucytosine	1
4106860960	c[nH]c(N)c(c)F	Flucytosine	1
10561807	C[O-]	Dantrolene Sodium	1
494454189	C=NN(CC)C(N)=O	Dantrolene Sodium	1
1531048361	NC(N)=O	Dantrolene Sodium	1
1670510852	CN=C([O-])CN	Dantrolene Sodium	1
1890668765	CC(=N)[O-]	Dantrolene Sodium	1
2089738716	CN(N)CC(=N)[O-]	Dantrolene Sodium	1
2254756311	C=NC(=O)N(C)N	Dantrolene Sodium	1
4225625916	CC([O-])=NC(N)=O	Dantrolene Sodium	1
26091732	COC(CN)C(C)O	Tobramycin	1
1409406604	CC(N)CC(C)O	Tobramycin	1
4049143819	CCC(N)C(O)O	Tobramycin	1
75208198	CC(O)CC(=N)Cl	acivicin	1
497936938	CC(O)C(N)C(=O)O	acivicin	1
1001929303	CCC(ON)C(C)N	acivicin	1
1675508538	CC(C)N	acivicin	1
1762828108	CON=C(C)Cl	acivicin	1
3938582833	C=NN(CC)C(=O)O	Furazolidone	1
2477227079	cc(C)cc(c)N	2-acetylaminofluorene (2-AAF)	1
2854154172	ccc(Cc)c(c)-c	2-acetylaminofluorene (2-AAF)	1
1480654374	cC1CC1(Cl)Cl	Ciprofibrate	1

3447654516	CC(C)(Cl)Cl	Ciprofibrate	1
994895109	CC(C)(S)S	Probucol	1
4164652423	cSC(C)(C)Sc	Probucol	1
526625799	cc(O)cc(c)O	Bambuterol	1
1844013313	NCCC(=O)[O-]	Calcium Pantothenate	1
99429666	CC(C)C	Capsaicin	1
900642759	CC=CCC	Capsaicin	1
948620800	C=CC(C)C	Capsaicin	1
1516788326	ccc(OC)c(c)O	Capsaicin	1
4205183701	CC=CC(C)C	Capsaicin	1
578168791	cc(c)COC	cerivastatin	1
676882998	cc(c)-c(c(c)C)c(c)C	cerivastatin	1
1170523770	cCOC	cerivastatin	1
1650575713	cnc(c(c)C)C(C)C	cerivastatin	1
1842405735	C=Cc(c(c)-c)c(C)n	cerivastatin	1
1989900410	cc(C)nc(c)C	cerivastatin	1
3102900200	cc(-c)c(CO)c(C)n	cerivastatin	1
40909315	CCCN(C)C	Mibefradil	1
370673334	cc(c)[nH]c(C)n	Mibefradil	1
2338012251	ccc(cc)C(C)C	Ciprofibrate	1
1286313644	cc(c)C1CC1(Cl)Cl	Ciprofibrate	1
598546392	cc(c)CCN	Melatonin	1
4190412450	cc(c)C(O)C(C)N	Phenylpropanolamine HCl	1
197860580	C=CC(CC)OC	Ethynodiol Diacetate	1
221686894	CC=C(CC)C(C)C	Ethynodiol Diacetate	1
718919707	CCCC(C)O	Ethynodiol Diacetate	1
3422364444	CC(=O)OC(C)C	Ethynodiol Diacetate	1
3660754788	CC(C)=CC(C)O	Ethynodiol Diacetate	1
4246621378	CCCC(C)C	Ethynodiol Diacetate	1
29318521	CNC(=O)CS	Cephapirin Sodium	1
2362099472	cc(c)SCC	Cephapirin Sodium	1
2462678498	cSCC(N)=O	Cephapirin Sodium	1
1293579910	cNC(=O)C(C)O	Iopamidol	1
1937645139	CC(O)C(N)=O	Iopamidol	1
140040439	C=NC(C)C(C)(C)C	Pinacidil	1
1195254652	CC(N)C(C)(C)C	Pinacidil	1
1442677706	CC(C)N	Pinacidil	1
1633980016	CN=C(NC)Nc	Pinacidil	1
1873979657	CC(C)N=C(N)N	Pinacidil	1
3235410551	cc(c)NC(=N)N	Pinacidil	1
440176060	cc(c)C(O)C(C)N	Methoxamine HCl	1
3662032074	ccc(c(c)O)C(C)O	Methoxamine HCl	1
2360467803	cc([nH])N	Flucytosine	1

2357881003	cc(N)[nH]c(n)=O	Flucytosine	1
355705209	cnc(=O)[nH]c	Flucytosine	1
3182087364	CC(O)CC#N	1-cyano-2-hydroxy-3-butene	1
2289927267	C=CCO	Allyl alcohol	1
1735430769	C=CCOC	allyl formate	1
1969557459	COC=O	allyl formate	1
2222711142	COC	allyl formate	1
2927823314	CCOC=O	allyl formate	1
4102492447	O=CO	allyl formate	1
721761124	NC(=O)C(O)(O)C(N)=O	Alloxan Hydrate	1
3111077996	CC(C)(O)O	Alloxan Hydrate	1
1633782713	cc(c)Cc(c)c	Hexachlorophene	1
1338531880	c[nH]cc(c)N	Uracil Mustard	1
1609817788	cc(c)N(CC)CC	Uracil Mustard	1
2126270858	c[nH]c(=O)c(c)N	Uracil Mustard	1
4055459154	CN(C)c(c[nH])c([nH])=O	Uracil Mustard	1
3806872436	cc(c)Sc(c)c	Bithionol	1
366759123	cc(Cl)cc(c)Cl	3,5-Dichloroaniline hydrochloride	1
2651257257	ccc(N)cc	3,5-Dichloroaniline hydrochloride	1
161656386	CC(C)S	Butoconazole Nitrate	1
430661433	CCC(Cn)Sc	Butoconazole Nitrate	1
595560908	cCCC(C)S	Butoconazole Nitrate	1
4140928901	C=CC(O)CC	1-cyano-2-hydroxy-3-butene	1
2405341397	C=CC(C)O	1-cyano-2-hydroxy-3-butene	1
2074443444	cc(c)S	Butoconazole Nitrate	1
1323026767	CCC#N	1-cyano-2-hydroxy-3-butene	1
1016271016	O=C(O)P(=O)(O)O	Foscarnet	1
1256623549	O=C(O)P	Foscarnet	1
1688640159	NC(CO)(CO)CO	Tromethamine	1
1026654242		Aurothioglucose	1
1741605201	CC(O)C(O)C(O)[S-]	Aurothioglucose	1
2448016348	C[S-]	Aurothioglucose	1
2702218056	COC([S-])C(C)O	Aurothioglucose	1
3234834868	CC(C)OC(C)[S-]	Aurothioglucose	1
4171844611	CC(O)[S-]	Aurothioglucose	1
2530051200	CCCN	Pamidronate	1
3562550253	NCCC(O)(P)P	Pamidronate	1
1218989618	[N+]CC(=O)O	Betaine HCl	1
1246877952	C[N+](C)(C)CC(=O)O	Betaine HCl	1
1371138585	O=P([O-])(O)OP(=O)(O)O	Citicoline	1
4028276449	COP(=O)([O-])OP	Citicoline	1
686051290	C=C(CC)C(C)O	Nalmefene	1
1075888140	C=C(C)CCC	Nalmefene	1

2168984005	C=C(C)C(Oc)C(c)(C)C	Nalmefene	1
187141432	C=CC	1-cyano-2-hydroxy-3-butene	1
1747261343	cc(c)SC(C)C	Butoconazole Nitrate	1
2834815755	cn(c)CC(C)S	Butoconazole Nitrate	1
4017977253	ccc(c[nH])-c(c)c	Amrinone	1
1494614853		Bismuth Subsalicylate	1
3287408874	cc(c)C(=O)O[Bi]	Bismuth Subsalicylate	1
3832890613	CO[Bi]Oc	Bismuth Subsalicylate	1
3884880000	ccc(O[Bi])c(c)C	Bismuth Subsalicylate	1
4276996454	cc(c)O[Bi]O	Bismuth Subsalicylate	1
202304456	C=Nc	1-Naphthyl isothiocyanate	1
540566222	cN=C=S	1-Naphthyl isothiocyanate	1
745524459	C=S	1-Naphthyl isothiocyanate	1
862549652	C=Nc(cc)c(c)c	1-Naphthyl isothiocyanate	1
3383674944	ccc(c(c)N)c(c)c	1-Naphthyl isothiocyanate	1
3554450330	cc(c)N=C=S	1-Naphthyl isothiocyanate	1
3891750802	N=C=S	1-Naphthyl isothiocyanate	1
932550026	ccc(O)c(c)n	Oxyquinoline Sulfate	1
2388214209	ccc(cc)c(c)n	Oxyquinoline Sulfate	1
2866316531	cnc(c(c)O)c(c)c	Oxyquinoline Sulfate	1
533219900	c[nH]c(=O)c(c)N	Amrinone	1
1849238813	cc(-c)cc(c)N	Amrinone	1
2899128730	ccc(N)c([nH])=O	Amrinone	1
3072065726	cc[nH]c(c)=O	Amrinone	1
3265401503	c[nH]cc(c)-c	Amrinone	1
1662852442	ccc(C(=O)O)c(c)O	Bismuth Subsalicylate	1
1037529607	cC(=O)O[Bi]O	Bismuth Subsalicylate	1
3562021113	cc(Cl)c(SC)c(c)Cl	Butoconazole Nitrate	1
429036415	O[Bi]O	Bismuth Subsalicylate	1
1301991264	cc(c)COC	Miconazole	1
2596116113	ccc(CO)c(c)Cl	Miconazole	1
778690921	cc([N+])cc(c)[N+]	1,3-dinitrobenzene	1
1134197127	cc(c)COC	Econazole Nitrate	1
1852035695	ccc(cc)CS	Sulconazole Nitrate	1
2089616642	cc(c)C(Cn)SC	Sulconazole Nitrate	1
2151770660	cCSC(C)c	Sulconazole Nitrate	1
3167560947	cc(c)CSC	Sulconazole Nitrate	1
3582414510	ccc(c(c)Cl)C(C)S	Sulconazole Nitrate	1
4209423444	cn(c)CC(c)S	Sulconazole Nitrate	1
1545850997	ccc(N=N)c(n)N	Phenazopyridine HCl	1
1845520275	cnc(N)c(c)N	Phenazopyridine HCl	1
4019310718	cccc(c)N	Phenazopyridine HCl	1
4100515081	cc(N)nc(c)N	Phenazopyridine HCl	1

4122441674	cccc(n)N	Phenazopyridine HCl	1
977029395	ccc(cc)S(C)=O	Sulfinpyrazone	1
1945468781	cc(c)S(=O)CC	Sulfinpyrazone	1
2000492875	CC(C)CCS	Sulfinpyrazone	1
2623496635	CCCS(c)=O	Sulfinpyrazone	1
371924701	CCCc(n)[nH]	Mibefradil	1
704989828	cnc(CC)[nH]c	Mibefradil	1
4235982241	cc(c)c(CC)c(c)c	3-Methylcholanthrene	1
2632990378	ccc(OC)c(c)Cl	indacrinone	1
2375728214	C=C(C)C(C)(CC)C(C)(C)C	Carbenoxolone Disodium	1
2569162574	CC(C)(C)C	Carbenoxolone Disodium	1
2734970704	CC(=O)[O-]	Carbenoxolone Disodium	1
3511282223	CC(C)C	Carbenoxolone Disodium	1
3562869593	CCCC(C)(C)C	Carbenoxolone Disodium	1
4133670410	CCC(OC)C(C)(C)C	Carbenoxolone Disodium	1
4172724256	CCCC(=O)[O-]	Carbenoxolone Disodium	1
4196713895	CCCC(C)(C)C	Carbenoxolone Disodium	1
1069305578	cc(c)CC(C)(c)C	indacrinone	1
2723621927	cc(C)c(C(C)=O)c(c)Cl	indacrinone	1
741655965	ccc(c(c)C)C(C)C	Mibefradil	1
2840260958	cc(C)c(Cl)c(c)Cl	indacrinone	1
3285398920	ccc(CC)c(c)C	indacrinone	1
3566563017	cc(c)C(=O)C(C)(C)c	indacrinone	1
4070567120	cc(c)C(C)(Cc)C(c)=O	indacrinone	1
1571441868	CCC(C)(CC)CO	Ciglitizone	1
2504847923	CC(C)(C)C	Ciglitizone	1
2741482242	cOCC(C)(C)C	Ciglitizone	1
60624089	cc(C)c(Cl)c(c)N	Meclofenamate Sodium	1
1237647219	ccc(C)c(c)Cl	Meclofenamate Sodium	1
2280294135	CCC(C)(CC)C(C)C	Carbenoxolone Disodium	1
2256698803	CC=C(C(C)C)C(C)(C)C	Carbenoxolone Disodium	1
2068609103	CC(=O)C(C(C)(C)C)C(C)(C)C	Carbenoxolone Disodium	1
1770582888	C=CC(=O)C(C)C	Carbenoxolone Disodium	1
3718710299	CN(C)S	Mezlocillin Sodium	1
3996271210	CN(C)C(=O)N(C)S	Mezlocillin Sodium	1
913183037	CCN(C(N)=O)C(N)=O	Azlocillin Sodium	1
2773790418	CNC(=O)N(C)C	Azlocillin Sodium	1
3160601726	CCNC(N)=O	Azlocillin Sodium	1
3377788910	CN(C)CCN	Azlocillin Sodium	1
367035058	cc(O)c(C(N)=O)c(c)O	Methicillin Sodium	1
845554841	ccc(Cl)c(c)C	Chlortetracycline HCl	1
1508213505	cc(c)C(C)(O)C(C)C	Chlortetracycline HCl	1
2164712624	cc(C)c(c(c)Cl)C(C)(C)O	Chlortetracycline HCl	1

3264663605	C=C(O)c(c(c)C)c(c)O	Chlortetracycline HCl	1
4262358	CC(C)(C)C	Carbenoxolone Disodium	1
12239218	CCC(C)(CC)C(=O)[O-]	Carbenoxolone Disodium	1
484125671	C=C(C)C(CC)C(C)(C)C	Carbenoxolone Disodium	1
494522277	CC(C)(C)C(=O)[O-]	Carbenoxolone Disodium	1
852377693	CC(=O)C=C(C)C	Carbenoxolone Disodium	1
984947543	CCC(C)(C(C)C)C(C)(C)C	Carbenoxolone Disodium	1
1050572201	CC(C)CC(C)(C)C	Carbenoxolone Disodium	1
1769392096	CCC(C)(C(C)C)C(C)C	Carbenoxolone Disodium	1
1680998488	ccc(C(=O)[O-])c(c)N	Meclofenamate Sodium	1
2383086756	cc(Cl)c(Nc)c(c)Cl	Meclofenamate Sodium	1
2470746019	cc(c)C(=O)[O-]	Meclofenamate Sodium	1
2222171723	cc(C)c(C(c)=O)n(c)C	Zomepirac	1
2843124472	cc(c)C(=O)c(c)n	Zomepirac	1
3089236829	cc(C)n	Zomepirac	1
3976507665	cc(C)n(C)c(c)C	Zomepirac	1
1742433703	CC=C(CC)C(C)N	Cephradine	1
1854136241	C=C(C)C	Cephradine	1
1902667082	C=C(C)C(N)C(N)=O	Cephradine	1
2056774955	CC=CCC	Cephradine	1
2405824091	C=CCC=C	Cephradine	1
2889988355	CCC=C(C)C	Cephradine	1
3643546905	C=CCC(=C)C	Cephradine	1
4117938018	CC=CCC	Cephradine	1
4124481675	CCC	Cephradine	1
4140259565	CCC	Cephradine	1
1847535815	ccc(cc)c(c)c	3-Methylcholanthrene	1
2060792449	ccc(c(c)c)c(c)c	3-Methylcholanthrene	1
2332506919	cc(C)c(c(c)C)c(c)c	3-Methylcholanthrene	1
2536221440	ccc(c(c)C)c(c)c	3-Methylcholanthrene	1
2681126977	cc(C)c(CC)c(c)c	3-Methylcholanthrene	1
2902363633	ccc(C)c(c)C	3-Methylcholanthrene	1
2228753047	ccc(C)c(C)n	Zomepirac	1
1096429503	cc(C)cc(C)n	Zomepirac	1
3198511505	COCC(C)C	Nisoldipine	1
92540367	ccc(CC)n(c)C	Zomepirac	1
1042181770	CCN(C)Cc	Nicardipine HCl	1
329345027	coc(=O)c(c)N	Novobiocin	1
498907842	COC(N)=O	Novobiocin	1
958505401	ccc(CC)c(c)O	Novobiocin	1
1231087734	cCC=C(C)C	Novobiocin	1
1524443508	cc(N)c(O)c(c)c	Novobiocin	1
734628039	CP(=O)(O)O	Foscarnet	1

1800485022	COC(C)C	Novobiocin	1
1908852800	CC(C)OC(N)=O	Novobiocin	1
2356591436	COC(C(C)O)C(C)O	Novobiocin	1
2498426181	cc(O)c(C)c(c)o	Novobiocin	1
2874253534	cc(c)OC(C)O	Novobiocin	1
3025621569	ccc(c(c)O)c(c)o	Novobiocin	1
3200566178	coc(c(c)C)c(c)c	Novobiocin	1
3316324715	cc(C)cc(c)C	Novobiocin	1
3467160439	cc(O)c(NC)c(=O)o	Novobiocin	1
3727705459	ccc(OC)c(c)C	Novobiocin	1
3975790142	C=CCc(c)c	Novobiocin	1
4054160989	cOC(OC)C(C)O	Novobiocin	1
3531344838	CS(N)(=O)=O	Mezlocillin Sodium	1
3278587996	NC(N)=O	Mezlocillin Sodium	1
1955620676	CCN(C(N)=O)S(C)(=O)=O	Mezlocillin Sodium	1
2195323342	CCC(C)(C(C)C)C(C)C	atractyloside	1
2399946206	CC(C)CC(C)(C)C	atractyloside	1
2526951897	CC(=O)OC(C)C	atractyloside	1
2627504773	CCC(CC)OC	atractyloside	1
2692193956	CC(O)CC(C)(C)C	atractyloside	1
2708632085	CC(O)C(OS)C(C)O	atractyloside	1
2925436761	CC(C)(C)C	atractyloside	1
3063557465	C=C(C(C)C)C(C)O	atractyloside	1
3217331490	COC(C(C)O)C(O)O	atractyloside	1
3367986192	C=C(C)C(O)C(C)(C)C	atractyloside	1
3455614769	CC(C)OC(C)O	atractyloside	1
3921411091	CCC(CC)(C(C)C)C(C)O	atractyloside	1
4055171359	CCC(C(C)C)C(C)(C)C	atractyloside	1
4272615500	CCC(C(C)(C)C)C(C)(C)C	atractyloside	1
847028072	CCN(CC)C(C)C	Bepridil HCl	1
1264267632	CC(N)CN(C)c	Bepridil HCl	1
1657871527	CN(C)C(CN)CO	Bepridil HCl	1
1832793247	cc(c)N(CC)Cc	Bepridil HCl	1
2524958857	COCC(C)C	Bepridil HCl	1
2562008222	CC(C)N	Bepridil HCl	1
2259415155	CCC(C)C	atractyloside	1
2112034852	CC(C)CC(=O)O	atractyloside	1
2915034670	COCC(C)N	Bepridil HCl	1
1632107681	CCCC(C)(C)C	atractyloside	1
1693576264	cCCCN	Mibefradil	1
1739921836	CC(C)(C)O	Mibefradil	1
1775038160	CCC(CC)(OC)C(C)c	Mibefradil	1
1937706626	CC(C)(O)CCN	Mibefradil	1

1962778678	CC(C)C(c)C	Mibefradil	1
2146682381	cCCC(C)(C)O	Mibefradil	1
2147351775	COCC(=O)O	Mibefradil	1
2573417174	cc(c)CCC	Mibefradil	1
2701060395	CCOC	Mibefradil	1
2874676774	cc(c)C(C(C)C)C(C)(C)O	Mibefradil	1
2992035385	CC(=O)OC(C)(C)C	Mibefradil	1
3914893947	CC(c)C	Mibefradil	1
4192068505	COC(=O)CO	Mibefradil	1
154580846	CCC(C(=O)O)C(C)C	atractyloside	1
449613684	C=C(C)C(CC)CC	atractyloside	1
631390493	CC(C)C(=O)O	atractyloside	1
633294005	CCC(=O)OC	atractyloside	1
954762907	CC(C)CC(C)O	atractyloside	1
1029762747	CC(C)O	atractyloside	1
2833025332	CCOCC	Bepridil HCl	1
2910246204	cc(C)cc(c)F	Flurbiprofen	1
486695774	CN(S)CCN	Mezlocillin Sodium	1
1866871746	CC[N+](C)(CC)CC	Oxyphenonium	1
1910338083	cc(O)c(OC)c(c)O	Gallamine Triethiodide	1
1294638265	COC(=O)N(C)C	Trimethadione	1
1675217462	CN(C)C(=O)C(C)(C)O	Trimethadione	1
3326389971	CC(C)(C)OC(N)=O	Trimethadione	1
3492576316	CC(=O)N(C)C(=O)O	Trimethadione	1
373346178	CC(C)C=C(Cl)Cl	Permethrin	1
476875975	CC1C(C(=O)O)C1(C)C	Permethrin	1
490325519	C=CC1C(C)C1(C)C	Permethrin	1
530852422	COC(=O)C(C)C	Permethrin	1
542715770	cc(c)COC	Permethrin	1
1241202526	C=C(Cl)Cl	Permethrin	1
1277913895	CC=C(Cl)Cl	Permethrin	1
1387249600	cCOC(C)=O	Permethrin	1
1445706287	cc(C)cc(c)O	Permethrin	1
1551769549	CC(C)C	Permethrin	1
1566670564	CC(C)C	Permethrin	1
3895551988	CC1C(C)C1(C)C	Permethrin	1
71136987	CN(C)S(C)(=O)=O	Mezlocillin Sodium	1
148887042	CCN(C(N)=O)C(N)=O	Mezlocillin Sodium	1
4075772858	C[N+](C)(C)CCO	Oxyphenonium	1
1491231198	C[N+](C)(C)C	Oxyphenonium	1
1041289540	CC(c)C(=O)[O-]	Fenoprofen Sodium	1
586114558	CCOC(C)=O	Oxyphenonium	1
2155827510	cc(c)C(C)C(=O)[O-]	Fenoprofen Sodium	1

270794601	CCCC(C)N	Gentamycin	1
463071824	CC(N)C(O)C(O)O	Gentamycin	1
513017850	COCC(C)(C)O	Gentamycin	1
541747943	CCC(OC)C(C)N	Gentamycin	1
775301426	CCC(N)C(O)O	Gentamycin	1
901080096	CNC(C)C	Gentamycin	1
1341416183	CNC(C)C(C)O	Gentamycin	1
1359300312	CNC(C)C	Gentamycin	1
1655430306	CC(C)N	Gentamycin	1
1764797296	CCOC(C)O	Gentamycin	1
2583740073	CC(C)N	Gentamycin	1
3106441609	CNC(C(C)O)C(C)(C)O	Gentamycin	1
3227493588	CC(C)OC(C)O	Gentamycin	1
3534064157	CC(N)C(C)(O)CO	Gentamycin	1
4093670086	CCCC(C)O	Gentamycin	1
1606071285	CC(C)C(C(C)C)C(C)O	Chenodiol	1
1901662236	CCC(O)C(C)C	Chenodiol	1
2217800772	CCC(CC)C(C)(C)C	Chenodiol	1
2844835166	NCCCN	desipramine	1

Table S2. The metrics of 10-fold stratified cross-validation over ten iterations.

ECFP4						
Iteration	CV	ACC	Loss	Total Size	DILI+	DILI-
1	1	0.567	0.673	94	47	47
1	2	0.767	0.526	94	48	46
1	3	0.767	0.526	94	48	46
1	4	0.767	0.494	94	48	46
1	5	0.900	0.299	94	48	46
1	6	0.900	0.299	94	48	46
1	7	0.900	0.299	94	48	46
1	8	0.900	0.299	94	48	46
1	9	1.000	0.227	94	48	46
1	10	1.000	0.227	94	48	46
2	1	0.467	0.716	94	47	47
2	2	0.733	0.544	94	48	46
2	3	0.733	0.409	94	48	46
2	4	0.800	0.373	94	48	46
2	5	0.867	0.250	94	48	46
2	6	0.867	0.250	94	48	46
2	7	0.933	0.218	94	48	46
2	8	0.900	0.187	94	48	46
2	9	1.000	0.171	94	48	46
2	10	1.000	0.171	94	48	46
3	1	0.533	0.669	94	47	47
3	2	0.833	0.407	94	48	46
3	3	0.833	0.367	94	48	46
3	4	0.967	0.274	94	48	46
3	5	0.967	0.237	94	48	46
3	6	0.967	0.237	94	48	46
3	7	1.000	0.192	94	48	46
3	8	1.000	0.192	94	48	46
3	9	1.000	0.192	94	48	46
3	10	1.000	0.126	94	48	46
4	1	0.633	0.703	94	47	47
4	2	0.767	0.449	94	48	46
4	3	0.867	0.288	94	48	46
4	4	0.967	0.208	94	48	46
4	5	0.933	0.177	94	48	46
4	6	0.933	0.177	94	48	46
4	7	0.933	0.177	94	48	46
4	8	0.967	0.107	94	48	46
4	9	0.967	0.107	94	48	46

4	10	0.967	0.107	94	48	46
5	1	0.600	0.666	94	47	47
5	2	0.767	0.396	94	48	46
5	3	0.933	0.224	94	48	46
5	4	1.000	0.217	94	48	46
5	5	0.967	0.194	94	48	46
5	6	0.967	0.194	94	48	46
5	7	0.967	0.184	94	48	46
5	8	0.967	0.128	94	48	46
5	9	0.967	0.128	94	48	46
5	10	0.967	0.128	94	48	46
6	1	0.433	0.700	94	47	47
6	2	0.867	0.505	94	48	46
6	3	0.833	0.367	94	48	46
6	4	0.967	0.276	94	48	46
6	5	0.933	0.253	94	48	46
6	6	0.967	0.190	94	48	46
6	7	0.967	0.190	94	48	46
6	8	1.000	0.122	94	48	46
6	9	1.000	0.122	94	48	46
6	10	1.000	0.084	94	48	46
7	1	0.500	0.696	94	47	47
7	2	0.500	0.696	94	48	46
7	3	0.700	0.624	94	48	46
7	4	0.867	0.418	94	48	46
7	5	0.767	0.367	94	48	46
7	6	0.933	0.307	94	48	46
7	7	0.933	0.307	94	48	46
7	8	0.933	0.307	94	48	46
7	9	0.933	0.254	94	48	46
7	10	1.000	0.186	94	48	46
8	1	0.533	0.690	94	47	47
8	2	0.800	0.498	94	48	46
8	3	0.900	0.226	94	48	46
8	4	0.900	0.226	94	48	46
8	5	0.933	0.225	94	48	46
8	6	0.933	0.205	94	48	46
8	7	0.933	0.178	94	48	46
8	8	1.000	0.147	94	48	46
8	9	1.000	0.147	94	48	46
8	10	1.000	0.110	94	48	46
9	1	0.400	0.716	94	47	47
9	2	0.800	0.476	94	48	46

9	3	0.833	0.339	94	48	46
9	4	0.900	0.281	94	48	46
9	5	0.833	0.270	94	48	46
9	6	0.867	0.242	94	48	46
9	7	0.867	0.242	94	48	46
9	8	0.967	0.188	94	48	46
9	9	1.000	0.145	94	48	46
9	10	1.000	0.126	94	48	46
10	1	0.500	0.677	94	47	47
10	2	0.500	0.677	94	48	46
10	3	0.500	0.677	94	48	46
10	4	0.733	0.472	94	48	46
10	5	0.800	0.395	94	48	46
10	6	0.967	0.225	94	48	46
10	7	0.967	0.225	94	48	46
10	8	0.967	0.225	94	48	46
10	9	0.967	0.225	94	48	46
10	10	0.967	0.225	94	48	46

ECFP6						
Iteration	CV	ACC	Loss	Total Size	DILI+	DILI-
1	1	0.400	0.712	94	47	47
1	2	0.633	0.629	94	48	46
1	3	0.733	0.508	94	48	46
1	4	0.833	0.347	94	48	46
1	5	0.900	0.318	94	48	46
1	6	0.900	0.318	94	48	46
1	7	0.900	0.318	94	48	46
1	8	1.000	0.191	94	48	46
1	9	1.000	0.191	94	48	46
1	10	1.000	0.191	94	48	46
2	1	0.400	0.723	94	47	47
2	2	0.767	0.489	94	48	46
2	3	0.900	0.273	94	48	46
2	4	0.900	0.273	94	48	46
2	5	0.900	0.273	94	48	46
2	6	1.000	0.165	94	48	46
2	7	1.000	0.165	94	48	46
2	8	1.000	0.165	94	48	46
2	9	1.000	0.165	94	48	46
2	10	1.000	0.087	94	48	46
3	1	0.533	0.689	94	47	47

3	2	0.800	0.424	94	48	46
3	3	0.967	0.162	94	48	46
3	4	1.000	0.123	94	48	46
3	5	1.000	0.091	94	48	46
3	6	1.000	0.091	94	48	46
3	7	1.000	0.091	94	48	46
3	8	1.000	0.072	94	48	46
3	9	1.000	0.072	94	48	46
3	10	1.000	0.042	94	48	46
4	1	0.500	0.678	94	47	47
4	2	0.500	0.678	94	48	46
4	3	0.800	0.440	94	48	46
4	4	0.900	0.287	94	48	46
4	5	0.933	0.210	94	48	46
4	6	0.933	0.165	94	48	46
4	7	0.933	0.165	94	48	46
4	8	0.933	0.149	94	48	46
4	9	0.967	0.138	94	48	46
4	10	1.000	0.099	94	48	46
5	1	0.467	0.683	94	47	47
5	2	0.767	0.396	94	48	46
5	3	0.900	0.237	94	48	46
5	4	0.967	0.141	94	48	46
5	5	0.967	0.118	94	48	46
5	6	1.000	0.091	94	48	46
5	7	1.000	0.091	94	48	46
5	8	1.000	0.083	94	48	46
5	9	1.000	0.083	94	48	46
5	10	1.000	0.058	94	48	46
6	1	0.633	0.700	94	47	47
6	2	0.867	0.310	94	48	46
6	3	0.967	0.177	94	48	46
6	4	0.967	0.141	94	48	46
6	5	0.967	0.118	94	48	46
6	6	0.967	0.118	94	48	46
6	7	1.000	0.101	94	48	46
6	8	1.000	0.070	94	48	46
6	9	1.000	0.070	94	48	46
6	10	1.000	0.070	94	48	46
7	1	0.467	0.717	94	47	47
7	2	0.833	0.364	94	48	46
7	3	0.867	0.241	94	48	46
7	4	1.000	0.146	94	48	46

7	5	0.967	0.133	94	48	46
7	6	0.967	0.133	94	48	46
7	7	0.967	0.133	94	48	46
7	8	0.967	0.133	94	48	46
7	9	1.000	0.091	94	48	46
7	10	1.000	0.067	94	48	46
8	1	0.433	0.707	94	47	47
8	2	0.833	0.423	94	48	46
8	3	0.900	0.300	94	48	46
8	4	0.967	0.196	94	48	46
8	5	0.933	0.173	94	48	46
8	6	0.933	0.173	94	48	46
8	7	1.000	0.106	94	48	46
8	8	1.000	0.106	94	48	46
8	9	1.000	0.104	94	48	46
8	10	1.000	0.064	94	48	46
9	1	0.433	0.690	94	47	47
9	2	0.767	0.459	94	48	46
9	3	0.933	0.281	94	48	46
9	4	0.933	0.197	94	48	46
9	5	0.933	0.163	94	48	46
9	6	1.000	0.113	94	48	46
9	7	1.000	0.113	94	48	46
9	8	1.000	0.113	94	48	46
9	9	1.000	0.083	94	48	46
9	10	1.000	0.071	94	48	46
10	1	0.633	0.665	94	47	47
10	2	0.633	0.665	94	48	46
10	3	0.700	0.529	94	48	46
10	4	0.967	0.161	94	48	46
10	5	0.967	0.161	94	48	46
10	6	0.967	0.161	94	48	46
10	7	0.967	0.135	94	48	46
10	8	0.967	0.103	94	48	46
10	9	0.967	0.103	94	48	46
10	10	0.967	0.097	94	48	46

FCFP4						
Iteration	CV	ACC	Loss	Total Size	DILI+	DILI-
1	1	0.400	0.708	94	47	47
1	2	0.400	0.708	94	48	46
1	3	0.400	0.708	94	48	46

1	4	0.667	0.613	94	48	46
1	5	0.800	0.345	94	48	46
1	6	0.800	0.345	94	48	46
1	7	0.800	0.345	94	48	46
1	8	0.867	0.253	94	48	46
1	9	0.867	0.253	94	48	46
1	10	0.867	0.253	94	48	46
2	1	0.567	0.693	94	47	47
2	2	0.800	0.497	94	48	46
2	3	0.867	0.275	94	48	46
2	4	0.900	0.211	94	48	46
2	5	0.967	0.161	94	48	46
2	6	0.967	0.161	94	48	46
2	7	0.967	0.128	94	48	46
2	8	1.000	0.088	94	48	46
2	9	1.000	0.088	94	48	46
2	10	1.000	0.062	94	48	46
3	1	0.433	0.697	94	47	47
3	2	0.767	0.405	94	48	46
3	3	0.900	0.261	94	48	46
3	4	0.933	0.218	94	48	46
3	5	0.967	0.152	94	48	46
3	6	0.967	0.150	94	48	46
3	7	0.967	0.150	94	48	46
3	8	0.967	0.133	94	48	46
3	9	0.967	0.133	94	48	46
3	10	1.000	0.115	94	48	46
4	1	0.633	0.672	94	47	47
4	2	0.800	0.444	94	48	46
4	3	0.967	0.211	94	48	46
4	4	0.967	0.169	94	48	46
4	5	0.933	0.159	94	48	46
4	6	0.967	0.145	94	48	46
4	7	0.967	0.145	94	48	46
4	8	1.000	0.079	94	48	46
4	9	1.000	0.079	94	48	46
4	10	1.000	0.079	94	48	46
5	1	0.533	0.692	94	47	47
5	2	0.800	0.380	94	48	46
5	3	0.933	0.231	94	48	46
5	4	0.967	0.187	94	48	46
5	5	1.000	0.141	94	48	46
5	6	1.000	0.139	94	48	46

5	7	1.000	0.139	94	48	46
5	8	1.000	0.090	94	48	46
5	9	1.000	0.090	94	48	46
5	10	1.000	0.079	94	48	46
6	1	0.600	0.672	94	47	47
6	2	0.833	0.392	94	48	46
6	3	0.967	0.227	94	48	46
6	4	0.967	0.197	94	48	46
6	5	1.000	0.146	94	48	46
6	6	1.000	0.146	94	48	46
6	7	1.000	0.110	94	48	46
6	8	1.000	0.081	94	48	46
6	9	1.000	0.081	94	48	46
6	10	1.000	0.057	94	48	46
7	1	0.667	0.651	94	47	47
7	2	0.933	0.278	94	48	46
7	3	0.933	0.226	94	48	46
7	4	0.967	0.177	94	48	46
7	5	0.967	0.174	94	48	46
7	6	0.967	0.174	94	48	46
7	7	0.967	0.144	94	48	46
7	8	1.000	0.077	94	48	46
7	9	1.000	0.077	94	48	46
7	10	1.000	0.065	94	48	46
8	1	0.500	0.691	94	47	47
8	2	0.733	0.493	94	48	46
8	3	0.867	0.289	94	48	46
8	4	0.900	0.202	94	48	46
8	5	0.967	0.170	94	48	46
8	6	1.000	0.151	94	48	46
8	7	1.000	0.151	94	48	46
8	8	1.000	0.072	94	48	46
8	9	1.000	0.072	94	48	46
8	10	1.000	0.072	94	48	46
9	1	0.533	0.677	94	47	47
9	2	0.800	0.407	94	48	46
9	3	0.933	0.212	94	48	46
9	4	0.933	0.212	94	48	46
9	5	1.000	0.176	94	48	46
9	6	0.933	0.173	94	48	46
9	7	1.000	0.148	94	48	46
9	8	1.000	0.100	94	48	46
9	9	1.000	0.100	94	48	46

9	10	0.967	0.093	94	48	46
10	1	0.600	0.682	94	47	47
10	2	0.833	0.442	94	48	46
10	3	0.967	0.222	94	48	46
10	4	0.967	0.188	94	48	46
10	5	0.967	0.188	94	48	46
10	6	1.000	0.142	94	48	46
10	7	1.000	0.111	94	48	46
10	8	1.000	0.097	94	48	46
10	9	1.000	0.097	94	48	46
10	10	1.000	0.069	94	48	46

			FCFP6			
Iteration	CV	ACC	Loss	Total Size	DILI+	DILI-
1	1	0.567	0.680	94	47	47
1	2	0.567	0.680	94	48	46
1	3	0.567	0.680	94	48	46
1	4	0.800	0.461	94	48	46
1	5	0.933	0.314	94	48	46
1	6	0.933	0.314	94	48	46
1	7	0.933	0.314	94	48	46
1	8	0.900	0.301	94	48	46
1	9	0.900	0.301	94	48	46
1	10	0.967	0.253	94	48	46
2	1	0.700	0.620	94	47	47
2	2	0.967	0.251	94	48	46
2	3	0.933	0.224	94	48	46
2	4	1.000	0.152	94	48	46
2	5	1.000	0.152	94	48	46
2	6	1.000	0.149	94	48	46
2	7	1.000	0.088	94	48	46
2	8	1.000	0.063	94	48	46
2	9	1.000	0.063	94	48	46
2	10	1.000	0.063	94	48	46
3	1	0.433	0.706	94	47	47
3	2	0.733	0.497	94	48	46
3	3	0.900	0.277	94	48	46
3	4	0.900	0.277	94	48	46
3	5	0.967	0.220	94	48	46
3	6	0.967	0.171	94	48	46
3	7	0.967	0.171	94	48	46
3	8	0.967	0.126	94	48	46

3	9	1.000	0.109	94	48	46
3	10	1.000	0.109	94	48	46
4	1	0.467	0.696	94	47	47
4	2	0.867	0.389	94	48	46
4	3	0.933	0.238	94	48	46
4	4	0.933	0.174	94	48	46
4	5	1.000	0.075	94	48	46
4	6	1.000	0.075	94	48	46
4	7	1.000	0.067	94	48	46
4	8	1.000	0.052	94	48	46
4	9	1.000	0.052	94	48	46
4	10	1.000	0.052	94	48	46
5	1	0.333	0.731	94	47	47
5	2	0.767	0.575	94	48	46
5	3	0.867	0.346	94	48	46
5	4	0.967	0.209	94	48	46
5	5	1.000	0.173	94	48	46
5	6	1.000	0.173	94	48	46
5	7	1.000	0.144	94	48	46
5	8	1.000	0.117	94	48	46
5	9	1.000	0.117	94	48	46
5	10	1.000	0.082	94	48	46
6	1	0.500	0.689	94	47	47
6	2	0.833	0.388	94	48	46
6	3	0.933	0.261	94	48	46
6	4	0.933	0.226	94	48	46
6	5	0.967	0.146	94	48	46
6	6	0.967	0.146	94	48	46
6	7	0.967	0.146	94	48	46
6	8	1.000	0.094	94	48	46
6	9	1.000	0.094	94	48	46
6	10	1.000	0.094	94	48	46
7	1	0.467	0.693	94	47	47
7	2	0.800	0.492	94	48	46
7	3	0.867	0.300	94	48	46
7	4	0.967	0.151	94	48	46
7	5	1.000	0.108	94	48	46
7	6	1.000	0.108	94	48	46
7	7	1.000	0.108	94	48	46
7	8	1.000	0.098	94	48	46
7	9	1.000	0.098	94	48	46
7	10	1.000	0.076	94	48	46
8	1	0.567	0.673	94	47	47

8	2	0.800	0.435	94	48	46
8	3	0.967	0.208	94	48	46
8	4	0.933	0.170	94	48	46
8	5	0.933	0.170	94	48	46
8	6	0.933	0.170	94	48	46
8	7	1.000	0.125	94	48	46
8	8	0.967	0.084	94	48	46
8	9	0.967	0.084	94	48	46
8	10	1.000	0.073	94	48	46
9	1	0.567	0.679	94	47	47
9	2	0.700	0.517	94	48	46
9	3	0.900	0.312	94	48	46
9	4	0.967	0.182	94	48	46
9	5	0.967	0.143	94	48	46
9	6	0.967	0.143	94	48	46
9	7	1.000	0.127	94	48	46
9	8	0.967	0.100	94	48	46
9	9	1.000	0.088	94	48	46
9	10	1.000	0.088	94	48	46
10	1	0.567	0.663	94	47	47
10	2	0.733	0.486	94	48	46
10	3	0.933	0.220	94	48	46
10	4	0.967	0.173	94	48	46
10	5	0.967	0.144	94	48	46
10	6	0.967	0.144	94	48	46
10	7	1.000	0.119	94	48	46
10	8	1.000	0.071	94	48	46
10	9	1.000	0.071	94	48	46
10	10	1.000	0.071	94	48	46

Table S3. Tanimoto distances between the same DILI classes.

Val DILI with Train DILI	Val No-DILI with Train No-DILI
0.30233	0.55556
0.16667	0.30769
0.20513	0.30000
0.10526	0.62963
0.18919	0.39394
0.39394	0.52632
0.19355	0.34694
0.35000	0.64516
0.65000	0.16000
0.26471	0.30000
0.30000	0.29412
0.21739	0.25000
0.25000	0.23810
0.27778	0.19403
0.36111	0.54545
0.26316	0.21212
0.28125	0.20000
0.66667	0.42105
0.28986	0.27273
0.72727	0.80556
0.20988	0.22857
0.49254	0.39623
0.32836	0.27692
0.61702	0.37500
0.76596	0.78333
0.40000	0.25301
0.31579	0.24074
0.48148	0.48889
0.23529	0.55556
0.37778	0.29412
0.57746	0.20455
0.26531	0.82000
0.25000	0.43750
0.47500	0.70968
0.76471	0.28125
0.47500	0.23000
0.66667	0.27143
0.26829	0.57143
0.18000	0.30435
0.50704	0.25610

0.53846
0.76190
0.35000
0.56452
0.39130
0.85246
0.87097
0.41791
0.45313
0.22321
0.23214
0.57692
0.45714
0.46429
0.62500
0.56452
0.20548
0.36634
0.52381
0.52941
0.33898
0.61765
0.27778
0.44737
0.25926
0.29630
0.26829
0.28788
0.25806
0.28333
0.40351
0.54098
0.33766
0.51724
0.34483
0.66667
0.60563
0.31667
0.29231
0.50505
0.36842
0.44898
0.88889

0.42466
0.22549
0.43077
0.25758
0.21839
0.23438
0.23077
0.20455
0.29000
0.47887
0.30526
0.31111
0.21538
0.86047
0.32258
0.65957
0.30303
0.41667
0.52941
0.18898
0.20000
0.26531
0.22472
0.28767
0.25000
0.18919
0.34286
0.61818
0.26316
0.25556
0.28571
0.25275
0.41860
0.32558

0.56044
0.50000
0.71429
0.64286
0.29268
0.43529
0.33898
0.21212
0.26230
0.08696
0.25000
0.26531
0.32558
0.33333
0.72414
0.20690
0.62069
0.21212
0.66000
0.07143
0.43421
0.68293

Table S4. The evauluation results on the 4 external datasets at all endurance levels.

(1) The entire Liew dataset

Level	Data Size	Neg Size	Pos Size	Accuracy	Sensitivity	Specificity	Balanced Accruacy	F1	AUC	Precision
0%	114	46	68	0.789	0.838	0.717	0.778	0.826	0.853	0.814
5%	120	49	71	0.783	0.845	0.694	0.769	0.822	0.839	0.800
10%	131	56	75	0.748	0.827	0.643	0.735	0.790	0.822	0.756
15%	146	65	81	0.705	0.802	0.585	0.694	0.751	0.811	0.707
20%	163	71	92	0.675	0.761	0.563	0.662	0.725	0.779	0.693
30%	177	79	98	0.650	0.745	0.532	0.638	0.702	0.756	0.664
100%	187	82	105	0.642	0.724	0.537	0.630	0.694	0.742	0.667

(2) valBLACK

Level	Data Size	Neg Size	Pos Size	Accuracy	Sensitivity	Specificity	Balanced Accruacy	F1	AUC	Precision
0%	38	16	22	0.974	0.955	1.000	0.977	0.977	0.955	1.000
5%	38	16	22	0.974	0.955	1.000	0.977	0.977	0.955	1.000
10%	38	16	22	0.974	0.955	1.000	0.977	0.977	0.955	1.000
15%	39	17	22	0.949	0.955	0.941	0.948	0.955	0.952	0.955
20%	41	19	22	0.902	0.955	0.842	0.898	0.913	0.947	0.875
30%	46	24	22	0.826	0.955	0.708	0.831	0.840	0.943	0.750
100%	47	24	23	0.830	0.957	0.708	0.832	0.846	0.937	0.759

(3) valPAIR

Level	Data Size	Neg Size	Pos Size	Accuracy	Sensitivity	Specificity	Balanced Accruacy	F1	AUC	Precision
0%	14	7	7	0.500	0.857	0.143	0.500	0.632	0.551	0.500
5%	14	7	7	0.500	0.857	0.143	0.500	0.632	0.551	0.500
10%	17	9	8	0.471	0.875	0.111	0.493	0.609	0.590	0.467
15%	18	10	8	0.500	0.875	0.200	0.538	0.609	0.631	0.467
20%	20	10	10	0.450	0.700	0.200	0.450	0.560	0.525	0.467
30%	20	10	10	0.450	0.700	0.200	0.450	0.560	0.525	0.467
100%	20	10	10	0.450	0.700	0.200	0.450	0.560	0.525	0.467

(4) valRANDOM

Level	Data Size	Neg Size	Pos Size	Accuracy	Sensitivity	Specificity	Balanced Accruacy	F1	AUC	Precision
0%	62	23	39	0.742	0.769	0.696	0.732	0.789	0.836	0.811
5%	68	26	42	0.735	0.786	0.654	0.720	0.786	0.810	0.786
10%	76	31	45	0.697	0.756	0.613	0.684	0.747	0.783	0.739
15%	89	38	51	0.640	0.725	0.526	0.626	0.698	0.759	0.673
20%	102	42	60	0.627	0.700	0.524	0.612	0.689	0.730	0.677

30%	111	45	66	0.613	0.682	0.511	0.596	0.677	0.700	0.672
100%	120	48	72	0.600	0.653	0.521	0.587	0.662	0.687	0.671

(5) Zhang dataset

Level	Data Size	Neg Size	Pos Size	Accuracy	Sensitivity	Specificity	Balanced Accruacy	F1	AUC	Precision
0%	80	27	53	0.950	1.000	0.852	0.926	0.964	0.957	0.930
5%	80	27	53	0.950	1.000	0.852	0.926	0.964	0.957	0.930
10%	82	28	54	0.951	1.000	0.857	0.929	0.964	0.957	0.931
15%	82	28	54	0.951	1.000	0.857	0.929	0.964	0.957	0.931
20%	82	28	54	0.951	1.000	0.857	0.929	0.964	0.957	0.931
30%	83	28	55	0.940	0.982	0.857	0.919	0.956	0.955	0.931
100%	85	28	57	0.941	0.982	0.857	0.920	0.957	0.952	0.933

(6) Ai dataset

Level	Data Size	Neg Size	Pos Size	Accuracy	Sensitivity	Specificity	Balanced Accruacy	F1	AUC	Precision
0%	84	21	63	0.881	0.905	0.810	0.857	0.919	0.920	0.934
5%	90	23	67	0.889	0.910	0.826	0.868	0.924	0.924	0.938
10%	95	26	69	0.895	0.913	0.846	0.880	0.926	0.925	0.940
15%	100	26	74	0.890	0.905	0.846	0.876	0.924	0.923	0.944
20%	109	26	83	0.890	0.904	0.846	0.875	0.926	0.919	0.949
30%	117	27	90	0.897	0.911	0.852	0.881	0.932	0.920	0.953
100%	121	27	94	0.893	0.904	0.852	0.878	0.929	0.911	0.955

(7) Kotsampasakou dataset

Level	Data Size	Neg Size	Pos Size	Accuracy	Sensitivity	Specificity	Balanced Accruacy	F1	AUC	Precision
0%	151	67	84	0.636	0.595	0.687	0.641	0.645	0.672	0.704
5%	235	98	137	0.600	0.591	0.612	0.602	0.633	0.664	0.681
10%	380	167	213	0.605	0.615	0.593	0.604	0.636	0.643	0.658
15%	530	242	288	0.596	0.635	0.550	0.593	0.631	0.620	0.627
20%	674	307	367	0.595	0.635	0.547	0.591	0.631	0.618	0.626
30%	865	400	465	0.594	0.634	0.548	0.591	0.627	0.618	0.620
100%	973	449	524	0.585	0.635	0.526	0.581	0.622	0.605	0.610
